# Age-Dependent Changes in Nrf2/Keap1 and Target Antioxidant Protein Expression Correlate to Lipoxidative Adducts, and Are Modulated by Dietary N-3 LCPUFA in the Hippocampus of Mice

**DOI:** 10.3390/antiox13020206

**Published:** 2024-02-06

**Authors:** Mario Díaz, Catalina Valdés-Baizabal, Daniel Pereda de Pablo, Raquel Marin

**Affiliations:** 1 Department of Physics, Faculty of Sciences, University of La Laguna, 38200 Tenerife, Spain; 2 Instituto Universitario de Neurociencias (IUNE), University of La Laguna, 38320 Tenerife, Spain; cvaldesb@ull.edu.es (C.V.-B.); dperedap@ull.edu.es (D.P.d.P.); rmarin@ull.edu.es (R.M.); 3 Laboratory of Cellular Neurobiology, Department of Basic Medical Sciences, Faculty of Health Sciences, University of La Laguna, 38200 Tenerife, Spain; 4 Associate Research Unit ULL-CSIC “Membrane Physiology and Biophysics in Neurodegenerative and Cancer Diseases”, 38200 Tenerife, Spain

**Keywords:** brain aging, hippocampus, Nrf2/Keap1, docosahexaenoic acid (DHA), heme-oxygenase 1 (HO-1), glutathione peroxidase 4 (GPx4), lipoxidative/nitrosative protein adducts

## Abstract

The brain has a high metabolism rate that may generate reactive oxygen and nitrogen species. Consequently, nerve cells require highly efficient antioxidant defenses in order to prevent a condition of deleterious oxidative stress. This is particularly relevant in the hippocampus, a highly complex cerebral area involved in processing superior cognitive functions. Most current evidence points to hippocampal oxidative damage as a causal effect for neurodegenerative disorders, especially Alzheimer’s disease. Nuclear factor erythroid-2-related factor 2/Kelch-like ECH-associated protein 1 (Nrf2/Keap1) is a master key for the transcriptional regulation of antioxidant and detoxifying systems. It is ubiquitously expressed in brain areas, mainly supporting glial cells. In the present study, we have analyzed the relationships between Nrf2 and Keap1 isoforms in hippocampal tissue in response to aging and dietary long-chain polyunsaturated fatty acids (LCPUFA) supplementation. The possible involvement of lipoxidative and nitrosative by-products in the dynamics of the Nrf2/Keap1 complex was examined though determination of protein adducts, namely malondialdehyde (MDA), 4-hydroxynonenal (HNE), and 3-nitro-tyrosine (NTyr) under basal conditions. The results were correlated to the expression of target proteins heme-oxygenase-1 (HO-1) and glutathione peroxidase 4 (GPx4), whose expressions are known to be regulated by Nrf2/Keap1 signaling activation. All variables in this study were obtained simultaneously from the same preparations, allowing multivariate approaches. The results demonstrate a complex modification of the protein expression patterns together with the formation of adducts in response to aging and diet supplementation. Both parameters exhibited a strong interaction. Noticeably, LCPUFA supplementation to aged animals restored the Nrf2/Keap1/target protein patterns to the status observed in young animals, therefore driving a “rejuvenation” of hippocampal antioxidant defense.

## 1. Introduction

The high metabolic demand of active neural areas is accompanied by the generation of either reactive oxygen (ROS) or nitrogen species (RNS). This phenomenon needs to be tightly controlled to avoid deleterious oxidative stress in tissue. Such regulation is particularly crucial for the maintenance and functioning of the hippocampus, a brain area mainly involved in learning and memory processing and consolidation. Highly efficient antioxidant systems (AOXs) collectively ensure a safe environment for both neuronal and glial functioning [1,2,3]. These systems are largely coordinated by active sensing of microenvironmental oxidative conditions, and mediated by transcriptional responses through nuclear factor erythroid-2-related factor 2 (Nrf2)/Kelch-like ECH-associated protein 1 (Keap1) [3,4,5].

Nrf2 is a broadly expressed transcription factor involved in the regulation of the cellular redox status. It is responsible for the activation of several antioxidants and phase I and II detoxifying and drug-metabolizing enzymes. In the absence of stimuli, Nrf2 protein remains predominantly in the cytoplasm where it interacts with the actin-binding protein Keap1. The Nrf2/Keap1 complex facilitates the inhibition of Nrf2 through ubiquitin ligase cullin3-mediated proteolysis [6,7,8,9]. When the levels of oxidants/stressors increase, Nrf2 unbinds from Keap1 and translocates into the nucleus, acting as a transcription factor. Nrf2 activates a series of genes containing antioxidant response elements (AREs) in their promoter regions, generating functional heterodimers containing a small MAF and other bZIP transcription factors. ARE-containing Nrf2-responsive genes include a battery of antioxidant and phase I and II detoxifying enzymes, such as hemeoxygenase-1 (HO-1), NAD(P)H quinone oxidoreductase 1 (NQO-1), members of the glutathione/glutaredoxin and thioredoxin/peroxiredoxin systems, superoxide dismutase, catalase, and drug transporters, among others [8,10,11].

Even though Nrf2/Keap1 is considered a ubiquitous pathway in the whole body, Nrf2 is poorly expressed in nerve cell populations, showing a preferential astrocytic ARE-mediated gene expression [12,13]. Both Nrf2 expression and Nrf2/Keap1/ARE signaling pathway activation decrease with age [11,14,15]. In correlation, aging is considered a key factor for increased oxidative stress in the brain. Age-related changes in the Nrf2 regulatory system involve upregulation of Keap1 and Bach1, which act as repressors of Nrf2 [4,6,9,16]. Apart from increased ROS/RNS and oxidative stress, Nrf2 reduction generates a lower capacity of injury-induced neurogenesis, autophagy deregulation, and ferroptosis, among other processes [3,14,17,18,19].

HO-1 and glutathione peroxidase 4 (GPx4) are two key players in preventing cell death in response to oxidative insults. These two proteins are physiologically pleiotropic, and their ablation leads to cell death and even embryonic lethality [14,20,21]. Both enzymes are largely complementary. Thus, whereas HO-1 is endowed with dual antioxidant and anti-inflammatory functions [22,23,24], GPx4 is capable of preserving membrane unsaturation and physicochemical stability of neuromembranes by directly acting on oxidized membrane phospholipids in the absence of phospholipase A2 [25,26,27].

Long-chain polyunsaturated fatty acids (LCPUFAs) play a crucial role in brain preservation along lifespan. In particular, docosahexaenoic acid (DHA) and arachidonic acid (AA) are both essential for a number of biological processes related to brain development and adaptability, neurophysiology, cellular signaling, intercellular communication, and superior cognitive tasks [28,29,30,31]. Unlike saturated or monounsaturated fatty acids, which can be synthesized locally, LCPUFAs have to be incorporated into the diet. Epidemiological studies have reported that LCPUFAs have a relevant role in neuroprotection against neurodegenerative pathologies such as Alzheimer’s disease and Parkinson’s disease, which are associated with pathological features such as mitochondrial dysfunction, neuroinflammation, and oxidative stress. Furthermore, different recent meta-analyses have emphasized the notion that omega-3 supplementation might have a positive effect on cognitive function during aging, which has put the focus on dietary n-3 LCPUFA as a nutraceutical strategy for therapeutic prevention of cognitive decline in elderly adults [32,33].

Several in vitro studies have shown that DHA, the most abundant and most polyunsaturated fatty acid in nerve cell phospholipids, may be involved in regulating redox systems. For instance, DHA can activate Nrf2 signaling and can modulate the expression of components from the glutathione and thioredoxin AOXs systems, through mechanisms that have not yet been characterized. As DHA is not a direct ligand of Nrf2, the question arises as to which endogenous ligands underlie the transcriptional responses elicited by DHA. ROS can oxidize DHA through non-enzymatic reactions that release highly electrophilic species, including 15-deoxy-Δ12,14-prostagandin, F4-neuroprostane and hydroperoxide aldehydes such as malondialdehyde (MDA) and 4-hydroxy-2-hexenal (HHE) [34,35,36,37]. Also, recent studies in neural and non-neuronal tissues have shown that DHA indirectly stimulates transcription of antioxidant and phase II detoxifying enzymes through activation of Nrf2 mediated by HHE [35,38,39]. These findings have been explained by HHE acting as an enhancer of DHA-induced transcriptional regulation [35,37,40,41]. Thus, AOXs transcription is enhanced by DHA, although other mechanisms not involving ARE-regulated genes may also be present. Recently, different signaling pathways, including peroxisome proliferator-activated receptors (PPAR)-α/γ, nuclear factor–kB (NF-kB), and MAPK cascade have been added to the spectrum of DHA-modulated transcriptional mediators of oxidative stress and other stress responses [37,42,43].

Despite the proven beneficial effects of DHA in the aged brain together with its potential neuroprotective effects against neurodegenerative diseases, the effects of long-term DHA administration in vivo on the Nrf2/Keap1 pathway and downstream proteins remain largely unexplored. In the present study, we have analyzed the combined effects of aging and dietary n-3 LCPUFA supplementation (mainly DHA) on the expression patterns of Nrf2, Keap1, HO-1, and GPx4 in the hippocampus of mice of different ages. As cellular Nrf2/Keap1 dynamics involve activation by electrophiles, including lipid-derived aldehydes, we have also determined the lipo/nitro-oxidative signature of protein adducts in hippocampal tissues in young and old animals, comparing the effects of n-3 LCPUFA administration. The complexity of the present experimental paradigm lays out the facts that (1) aging-associated changes are most often subtle and compensated by physiological redundancy, and (2) DHA peroxydability in the pro-oxidant brain environment makes it an active source for reactive lipoxidative compounds. Therefore, there must exist a precise window for non-enzymatic DHA transformation that, under physiological conditions during aging, allows discrimination between a neuroprotective antioxidant role and induction of mild oxidative stress. In the present study, the overall outcomes indicate a significant effect of aging on the expression of Nrf2/Keap1-HO-1/GPx4. Interestingly, dietary n-3 LCPUFA causes an age-dependent modulation of this signaling pathway, which partly restores the young phenotype in the hippocampus of older animals.

## 2. Materials and Methods

### 2.1. Animals and Experimental Groups

C57BL/6 mice were divided into four different groups as follows: 5–6 months old, fed with a standard diet (CTRL < 6 M); 5–6 months old, fed with an n-3 LCPUFA diet (LCPUFA < 6 M); 15 months old fed, with a standard diet (CTRL 15 M); and 15 months old, fed with an n-3 LCPUFA diet (LCPUFA 15 M). The animals subjected to the protocol were all nulliparous virgin female littermates, weaned and maintained together according to standard animal house conditions (12 h light–dark cycle and ad libitum access to food and water). All procedures were conducted in accordance with local and national guidelines (European Council Directive 86/602/EEC) and had been approved by the institutional Research Ethics and Animal Welfare Committee (CEIBA 2019-0346).

The standard diet provided was a Teklad Global 14% Protein Rodent Maintenance Diet by Envigo ad libitum. The LCPUFA groups (<6 M and 15 M) were initially fed with the standard diet until 6 and 12 weeks before sacrifice, respectively. Hence, the standard diet was replaced by one supplemented with Eupoly-3 DHA oil (Biosearch Life, Madrid, Spain) during manufacturing, containing the final (LCPUFA) diet, a 2.1:1 eicosapentaenoic acid (EPA)-to-DHA ratio enrichment (Supplementary Table S1). The same dietary administration schedule was used in our previous study aimed at analyzing the modulatory effects of LCPUFA on hippocampal dysfunction associated with aging. The nutritional content of the respective diets and the detailed schedule for administration can be found in Taoro-Gonzalez et al., 2022 [44]. Accordingly, the main difference between diets lay in the supplementation with 2.1:1 (0.56 mg EPA/kg diet: 0.26 mg DHA/kg diet) of the LCPUFA diet. As most EPA is readily converted to DHA in the liver, the main supply of LCPUFA to brain tissue will be in the form of DHA

### 2.2. Sample Preparation and Tissue Extracts

The experimental animals were sacrificed by cervical dislocation followed by decapitation. The hippocampi were rapidly dissected on ice, flash frozen in liquid nitrogen, and maintained at −80 °C until further processing. When the time came, hippocampal tissue was homogenized with a polytron (Kinematica, Switzerland) in cold homogenizing buffer composed of 66 mM Tris/HCl pH = 7.4, 35 mM NaCl, 1% SDS, 1 mM EGTA, 5 mM EDTA, 10% glycerol, 1 mM NaF, 1 mM Na_3_VO_4_, 1 mM MPSF, and 1x Complete Protease Inhibitor Cocktail. The samples were centrifuged at 13,000× *g* and 4 °C for 20 min. Supernatants were collected and stored at −20 °C until being used.

### 2.3. Western Blots

Proteins were resolved by SDS-PAGE. Approximately 75µg of total protein were mixed with 4x loading buffer (0.13 M Tris/HCl pH = 6.8; 2.1% SDS; 21% glycerol; 5.3% 2-mercaptoethanol and 20 mM bromophenol blue), charged into 12% polyacrylamide gels (456-1095, BioRad) and run in TGS buffer (1610772, BioRad) for 60 min. Then, proteins were transferred to PVDF membranes (170-4156, BioRad) using a Trans-Blot Turbo Transfer System (BioRad, Hercules, CA, USA). The membranes were blocked with everyBlot blocking buffer (12010020, Bio-Rad) for 5 min at RT, and incubated with corresponding antibodies overnight. Antibodies used were: anti-Nrf2 (ab137550, 1:500), anti-Keap1 (ab227828, 1:2000), anti-GPx4 (ab125066, 1:1000), anti-4-HNE (ab46545, 1:1000), anti-nitrotyrosine (NTyr, SC-32757, 1:1000), anti-MDA (STA031, 1:1000) anti HO-1 (ab189491, 1:2000) anti-ubiquitin (ab7254, 1:1000) and anti-tubulin (ab7792 and ab18251, 1:1000 both). Afterwards, membranes were washed with TBS-Tween buffer, incubated for 1 h at room temperature with 1/5000 anti-rabbit HRP-conjugated secondary antibody prepared in everyBlot blocking buffer, and washed again with TBST-Tween. The signal was revealed with a ClarityTMWestern ECL substrate (Bio-Rad). Band detection was performed with a Chemie-Doc MP Imaging System (Bio-Rad), and its optical density was analyzed using the Image Lab 6.0.1 software. Band intensities were normalized by referring to tubulin band intensity, which was also used as a protein loading control.

### 2.4. Statistics

All variables were initially assessed by one-way ANOVA followed by post hoc Tukey’s or Games–Howell tests for multiple comparisons between groups depending on homoscedasticity (Levene’s test) or non-parametric Kruskal–Wallis followed by Mann–Whitney U tests. Between-groups size effects were calculated using Cohen’s d [45,46,47]. The internal consistency of groups was assessed by Cronback’s alpha and intraclass correlation. Influences of main factors (age and diet) and their potential interactions were performed by two-way ANOVA, which included the calculus of partial Eta square (η_p_^2^) for the determination of effect sizes. Pearson’s and partial correlations, lineal regression, ANCOVA, and Cohen’s f^2^/R^2^ analyses [48] were performed to assess the statistical significances and effect sizes of bivariate relationships between different variables and between groups. Multivariate analyses were performed using hierarchical cluster analysis (HCA) to determine distances between groups, and linear discriminant function analysis (LDFA) to obtain the lowest number of discriminant canonical functions explaining the largest proportion of variance and the variables with the largest absolute correlation within each discriminant function (DF). This allowed the discrimination of experimental groups based on a linear combination of predictors (vectors) to maximize separation between projected data classes [49]. The SPSS 22.0 (IBM, New York, NY, USA) software package was used throughout. Some algorithms were implemented using Python on Excel data files.

## 3. Results and Discussion

### 3.1. Effects of Aging and Dietary n-3 LCPUFA Supplementation in Hippocampal Nrf2 Expression

**Figure 1:**
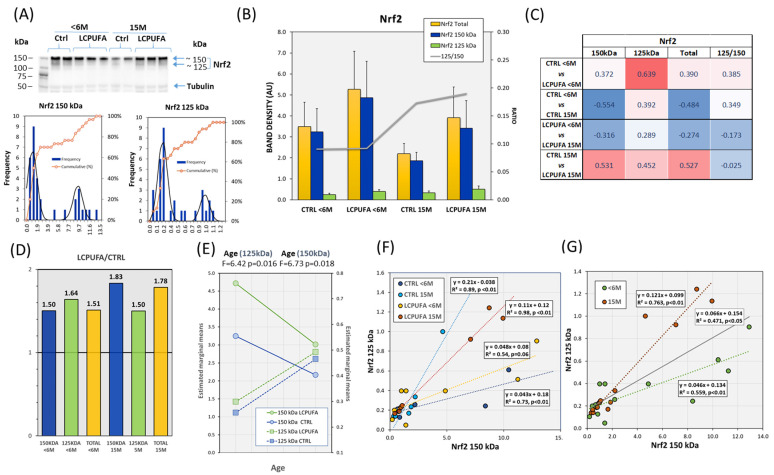
Effects of aging and LCPUFA treatment on the hippocampal expression of Nrf2. (**A**) Representative western blots of Nrf2 immunodetection in the four experimental groups showing the two Nrf2 bands. The lower panels show the frequency distribution of Nrf2 bands in the whole dataset. (**B**) Analyses of Nrf2 band intensities and band ratio in the four groups. (**C**) Analyses of effect sizes (Cohen’s d) for band intensities and ratios between individual groups. (**D**) Analyses of LCPUFA-to-CTRL ratios for the different Nrf2-age pairs. (**E**) Marginal means for age and dietary factors in the two-way ANOVA. The results indicated the presence of significant age-related changes but for diet or factor interaction. (**F**,**G**) Bivariate relationships for Nrf2 bands with lower molecular weight bands acting as dependent variable in the four experimental groups (**F**) and grouped by age (**G**) as inferred from the results in (**E**). Number of samples per group: 7 (CTRL < 6 M), 8 (LCPUFA < 6 M), 6 (CTRL 15 M), 9 (LCPUFA 15 M).

Nrf2 expression was determined in whole hippocampus extracts by western blotting. The results shown in Figure 1A indicate the presence of a main band migrating at 150 kDa and a secondary band at approximately 125 kDa. The two bands were identified in all experimental groups yet with different expression levels, in correlation with both age and LCPUFA factors. The distinct Nrf2 isoforms can be explained by alternative splicing generating different variants of the Nrf2 protein. The most common Nrf2 post-translational modification described is phosphorylation, although other modifications, such as acetylation, ubiquitination, sumoylation, and glycosylation have also been reported [11,50,51]. The extent and specific positioning of these modifications can differ, leading to variations in molecular weight observed in different studies. The post-translational modifications of Nrf2 allow distinct functional properties’ isoforms, protein stability, and subcellular distribution [11].

The relative abundance of both bands in the dataset were 90.3% (Nrf2 150 kDa) and 9.7% (Nrf2 125 kDa). Initial exploratory analyses of Nrf2 expression revealed unexpected bimodal distributions for the two molecular weight isoforms (Figure 1A,B), indicating a complex expression pattern of the transcription factor in the in vivo hippocampus under unstimulated conditions. The existence of such bimodal patterns led to considerable intragroup variability, which hampered the detection of significant differences between groups for either band or total Nrf2 under univariate statistical premises (Figure 1B). However, apparent differences between groups were evident. Therefore, we used an alternative ANOVA-like estimation of size effects [45,46,52]. The results indicate small-to-moderate effects of: (1) age on Nrf2 150 kDa (η_p_^2^ = 0.031) and Nrf2 125 kDa (η_p_^2^ = 0.022); and (2) diet on Nrf2 150 kDa (η_p_^2^ = 0.025) and Nrf2 125 kDa (η_p_^2^ = 0.032), which increase to moderate to large using the complementary isoform as covariate. Using Cohen’s d for comparison of size effects between groups (Figure 1C), we observed that aging caused a moderate reduction effect on Nrf2 150 kDa in CTRL and LCPUFA, which was followed by similar changes in total Nrf2, as opposed to Nrf2 125 kDa. These results are in line with the general agreement that in older organisms there is a decline of total Nrf2 protein and an age-related impairment of Nrf2/ARE function [11,14,15]. We conclude that hippocampal aging is accompanied by a reduction of total Nrf2 secondary to reduction of the most abundant 150 kDa isoform.

Moreover, the magnitude of age-related changes was smaller for LCPUFA than for CTRL groups of an identical age, indicating that Nrf2 expression is increased in LCPUFA groups. In agreement, analyses of the Nrf2 ratio 125/150 indicated that groups exposed to the n-3 LCPUFA supplemented diet exhibited increased band ratios compared to control groups at either age (Figure 1B line plot). In fact, intergroup comparisons for LCPUFA/CTRL ratios (Figure 1D) revealed that, on average, dietary treatment increased Nrf2 150 kDa (50.1–83.4%), Nrf2 125kDa (49.9–63.7%), and total Nrf2 (51.0–78.3%). These results strongly indicate a positive modulatory role of n-3 LCPUFA in the expression of the transcription factor. Further, covariance analyses indicate that aging is the main factor in detecting group differences. Indeed, irrespective of the diet, aged animals exhibited lower Nrf2 150 kDa and higher Nrf2 125 kDa expression levels as compared to young animals (Figure 1E). Aging also increased the 125/150 ratio in 15 M hippocampi (F = 6.402, *p* = 0.019). Another interesting finding was that the effect of the diet appeared to be dependent on the age of the mice. Indeed, as shown in Figure 1F,G, regression analyses for Nrf2 125 kDa as an independent variable vs. Nrf2 150 kDa as a dependent variable revealed significant positive relationships in nearly all groups (Figure 1F), as well as in the whole dataset (Figure 1G, R^2^ = 0.471; *p* < 0.05). A more detailed analysis showed significantly higher regression and correlation coefficients for 15 M groups as compared to young groups (Table 1B, Figure 1G). These data suggest that n-3 LCPUFA supplementation in aged animals increases hippocampal levels of total Nrf2, yet in an isoform-dependent manner. The meaning of these results indicates a complex regulation of Nrf2 in the in vivo hippocampus under unstimulated conditions, i.e., in the absence of acute oxidative stress.

### 3.2. Effects of Aging and Dietary n-3 LCPUFA Supplementation on Hippocampal Keap1 Expression

**Figure 2:**
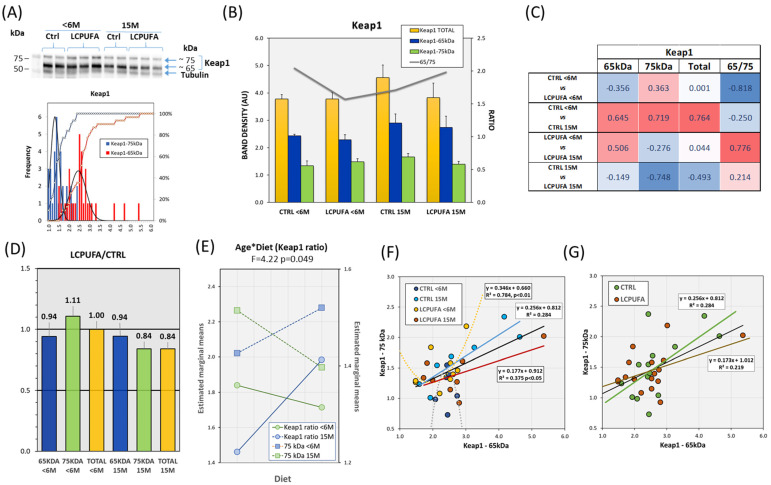
Effects of aging and LCPUFA treatment on the hippocampal expression of Keap1. (**A**) Representative western blots of Keap1 immunodetection in the four experimental groups showing two Keap1 bands migrating at 75 kDa and 65 kDa. The lower panels show the frequency distribution of Keap1 bands in the whole dataset. (**B**) Analyses of Keap1 band intensities in the four groups (bars) and the 65 kDa/75 kDa ratio (gray line). (**C**) Analyses of effect sizes (Cohen’s d) for band intensities and ratios between individual groups. (**D**) Analyses of LCPUFA-to-CTRL ratios for the different Keap1-age pairs. (**E**) Marginal means for age and dietary factors in the two-way ANOVA. The results indicated the presence of significant age–diet interaction for the Keap1 ratio. (**F**,**G**) Bivariate relationships for Keap1 bands with the higher molecular weight band acting as a dependent variable in the four experimental groups (**F**) and grouped by diet factor (**G**) as inferred from the results in (**E**). Number of samples per group: 6 (CTRL < 6 M), 9 (LCPUFA < 6 M), 7 (CTRL 15 M), 8 (LCPUFA 15 M).

In parallel with Nrf2, Keap1 expression was also determined in hippocampal extracts from the same animal samples. The results shown in Figure 2A indicate the presence of a main band migrating at approximately 65 kDa and a second higher molecular weight band detected at 75 kDa. The expression pattern of the two bands followed normal distributions (Figure 2A), with relative abundances of 63.8% (Keap1 65 kDa) and 36% (Keap1 75 kDa). One way ANOVA indicated that there were no differences between groups for neither total Keap1 content nor the 65 kDa/75 kDa proportion (Figure 2B). However, an estimation of size effects for comparisons between groups using Cohen’s d (Figure 2C) indicates moderate to large differences between CTRL <6 M and 15 M groups for Keap1 65 kDa, Keap1 75 kDa, and total Keap1. In contrast, the Keap1 band pattern was not reproduced in LCPUFA samples with the exception of 65 kDa isoform.

Unlike the Nrf2 results, the LCPUFA/CTRL ratios calculated for each isoform and group indicate the absence of important differences (Figure 2D), except for the 75 kDa band which was 26.7% higher in <6 M mice than in the 15 M group. Two-way ANOVA revealed a significant interaction between age and diet factors for the Keap1 ratio, which was partly due to a slight interaction between main factors in Keap1 75 kDa (Figure 2E). ANOVA-like estimation of size effects indicated a small to moderate effect for both age on Keap1 65 kDa (η_p_^2^ = 0.023) and age*diet interaction for Keap1 75 kDa (η_p_^2^ = 0.023). This was reflected in a very large size effect of age*diet interaction for 65 kDa/75 kDa ratio (η_p_^2^ = 0.109) detected in Figure 2E. Collectively, these findings support the existence of a direct age and diet interaction in the expression pattern of Keap1 isoforms.

Correlation analyses on the whole dataset indicate that the two bands were positively associated (r = 0.45 *p* < 0.01; Table 1A). Noticeably, this relationship was not significant using age and diet as control variables in the partial correlation analyses. This was because the two bands were only significantly correlated in the 15 M group (Table 1B,C). In line with this, regression analyses on Keap1 65 kDa (independent variable) and Keap1 75 kDa (dependent variable) showed that the two bands were linearly related in the whole dataset (β = 0.256, R^2^ = 0.284, *p* < 0.1). However, this was secondary to the significant association in the 15 M groups, where regression coefficients were higher (nearly two-fold) for the CTRL group (Figure 2F). Confirmatory was the fact that regression lines were significant for both dietary conditions, independent of the animal’s age (Figure 2F).

### 3.3. Effects of Aging and Dietary n-3 LCPUFA Supplementation on Nrf2-Keap1 Association

Given the responses to diet and aging of Nrf2 and Keap1 in the same hippocampal samples, we next explored their potential relationships in the four experimental groups. We first performed Pearson’s and partial correlation analyses for the influences of diet and aging on the coexpression Nrf2 and Keap1 (Table 1). Significant correlation coefficients were observed for Nrf2 variables (the 150 kDa and 125 kDa bands and Nrf2 total) and Keap1 65 kDa in the whole dataset (Table 1A). A similar analysis was performed for diet and age variables (Table 1B,C). In all the cases, bivariate relationships displayed negative correlation coefficients (Table 1).

In contrast, Keap1 75 kDa was positively related to total Nrf2 but not to Nrf2 150 kDa or Nrf2 125 kDa, except for the control diet (with Nrf2 150 kDa, *r* = 0.69, *p* < 0.05). This indicates a regulatory role of age and diet in the interaction of Keap1 75 kDa with Nrf2 isoforms, as revealed by the results of partial correlation analyses shown in Table 1A. Surprisingly, neither Nrf2 variable appeared to be related to total Keap1 in the whole dataset or under the effect of main factors (Table 1). The reason for this discrepancy is that Keap1 isoforms (65 and 75 kDa) interact oppositely with Nrf2 isoforms (opposed correlation coefficients), which are unmasked in the partial correlation analyses (Table 1A).

Nuanced functional isoforms of Keap1 exhibiting different molecular weights is not unprecedented [53,54,55,56]. Previous experiments indicated that Keap1 comprises two isoforms (α and β) which were localized in the cytoplasmic compartments but differed in their N-terminal sequence (amino acids 1 and 32) yet conserving their Ketch/DGR and C-terminal domains unchanged. The two isoforms of Keap1 (α and β) regulate the expression abundance of Nrf2 and its function on target genes HO-1 and NQO-1 [6] by interaction with the N-terminal Neh2 domain of Nrf2 to control the ubiquitin-mediated proteasomal degradation pathway [7].

Although Keap1 isoforms function as substrate adaptors to bind the CUL3-RBX1 E3 ubiquitin ligase complex, for the negative regulation of Nrf2, transcriptome sequencing and experimental approaches have emerged that differentially affect the expression of cell cycle- and apoptosis-related genes, possibly regulated by modulating PTEN signaling to the PI3K-mTOR pathway [53].

**Figure 3:**
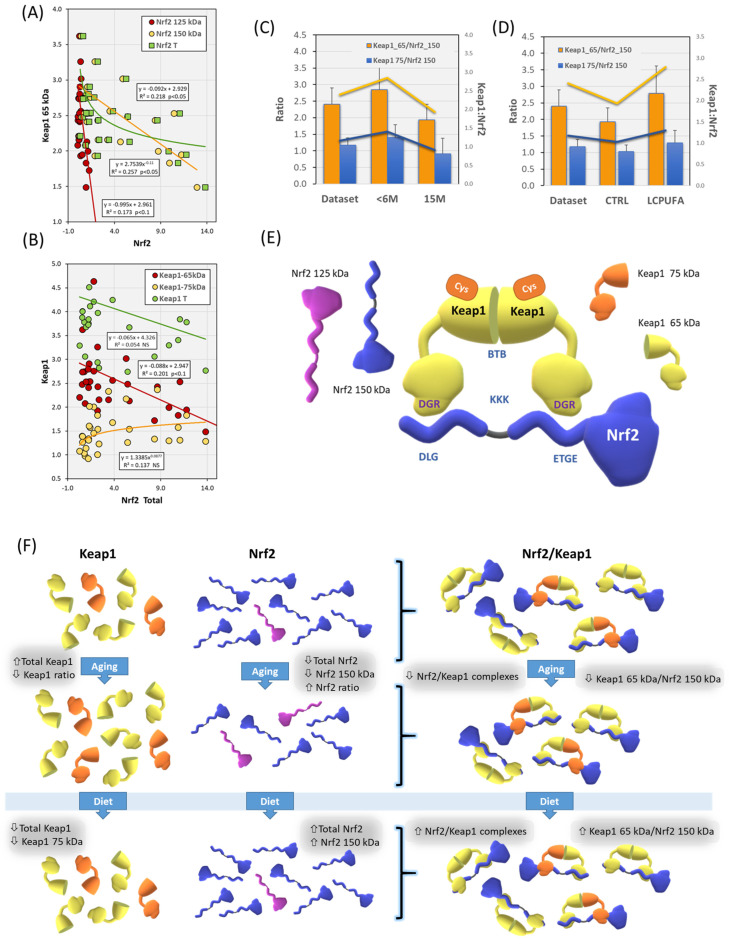
Summary of Keap1—Nrf2 relationships in hippocampal extracts. (**A**) Regression analyses for short Keap1 isoforms over Nrf2 isoforms and total Nrf2. (**B**) Similar regression analyses but reflecting changes in Keap1 isoforms and total Keap1 as a function of total Nrf2. Regression equations are indicated for each variable together with the determination coefficient (R^2^) and the statistical significance of the regression coefficient. (**C**,**D**) Analyses of Keap1 to Nrf2 ratios in the dataset and as a function of age (**C**) and diet (**D**) factors. (**E**) Schematic representation of the basic structure of the Keap1/Nrf2 complex. The different interactions of Keap1/Nrf2 motifs are indicated (see text). KKK: lysine-rich motif for ubiquitin conjugation. (**F**) A sketch summarizing the changes in the amounts and distributions of Keap1 and Nrf2 isoforms in the hippocampus in response to aging (Aging), and in old animals in response to n-3 LCPUFA diet.

Besides correlation analyses, regression analyses allow a deeper exploration of the degree of influence of the main factors on Nrf2 and Keap1 expression interplay (Table 1). Indeed, very different regression coefficients were observed for Nrf2 150 kDa and Nrf2 125 kDa over Keap1 65 kDa (10 times larger for Nrf2 125 kDa than for Nrf 150 kDa, *p* < 0.005) (Figure 3A). These differences changed the relationship between Keap1 65 kDa and total Nrf2 to a negative exponential with a decay constant of 0.126 (Figure 3A). The analyses using Nrf2 150 kDa as an independent variable showed no correlation with total Keap1, but oppositely related to 65 kDa and 75 kDa Keap1 isoforms (Figure 3B). These data agree with the previous hypothesis that Keap1 proteins might display complementary interactions with Nrf2 isoforms (Table 1A).

Finally, from the examination of the slope ratio (Figure 3C,D), the possible stoichiometry of Keap1 to Nrf2 subunits may be roughly estimated, being 2:1 for Keap1 65 kDa:Nrf2 150 kDa and 1:1 for Keap1 75 kDa:Nrf2 150 kDa in the whole dataset. Further, while the stoichiometry for Keap1 75 kDa did not change in response to aging or dietary treatment on their own, the ratio approached 3:1 for Keap1 65 kDa in young animals and those exposed to the LCPUFA diet (Figure 3C,D).

Of note, these statistical stoichiometry estimations were in close agreement with the 2:1 values reported for the Keap1:Nrf2 complex, where Keap1 forms a dimer through its N-terminal BTB domain [5,56]. Accordingly, a single Nrf2 protein binds to the Kelch domain of one member of the Keap1 dimer through its ETGE motif and to the second member of the Keap1 dimer through its DLG motif [5,56]. Moreover, it has been proposed that the Nrf2/Keap1 complex adopts two different alternative conformations: open and closed [57]. The open conformation does not allow for Nrf2 ubiquitination and protects Nrf2 from proteasomal degradation. It is formed by newly synthesized Nrf2 bound to one Keap1 in the dimer via a high-affinity ETGE motif. In the closed configuration, the second member of the Keap1 dimer binds to the low-affinity DLG motif of Nrf2, which predisposes Nrf2 for Keap1/Cul3/E3 ligase-dependent polyubiquitination and subsequent proteasomal degradation [5,57]. A summary of the amounts and potential interactions between Keap1 and Nrf2 isoforms in the present paradigm of aging and dietary modulation is illustrated in Figure 3E,F.

Our present results indicate that aging and LCPUFA interactively modify the molecular availability of Nrf2 and Keap1 subunits as a potential mechanism to modulate their functionality in the absence of acute oxidative challenges. The relative abundance of two Keap1 isoforms suggests that the formation of a Keap1 homo/heterodimer interacting with Nrf2 might provide an adaptive and metabolically favorable strategy to regulate the dynamics of extranuclear export, ubiquitination, and proteasomal degradation [8,9,11]. This would provide potential mechanisms to control Nrf2-induced transcriptional activation under changing basal stages in response to aging.

**Figure 4:**
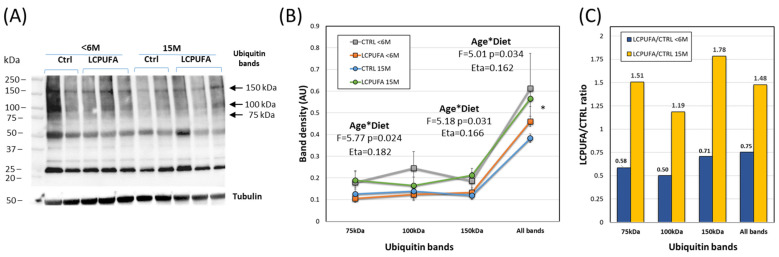
Effects of aging and LCPUFA treatment on the ubiquitination pattern of hippocampal extracts. (**A**) Representative western blot of ubiquitin immunodetection in the four experimental groups, showing the bands migrating at 75 kDa, 100 kDa, 105 kDa, and pooled data. (**B**) Analyses of immunoreactive ubiquitin bands (75, 100, 150, and pooled bands) in the four groups. (**C**) Analyses of LCPUFA-to-CTRL ratios for the different bands in young (<6 M) and aged (15 M) groups. * *p* < 0.05 between LCPUFA 15M or CTRL < 6 M and CTRL 15M. Number of samples per group: 6 (CTRL < 6 M), 9 (LCPUFA < 6 M), 7 (CTRL 15 M), 8 (LCPUFA 15 M).

In an attempt to detect extranuclear modifications of Nrf2 and Keap1 in association with the modulatory effects of LCPUFA on aging, we explored the ubiquitination pattern of hippocampal extracts. The results are shown in Figure 3. As expected, a complex pattern of immunoreactive bands was observed in the four experimental groups (Figure 4A). We have focused on bands that could match the isoforms identified for Nrf2 and Keap1 (app 75, 100, and 150 kDa) described above. We detected differences in the overall ubiquitination pattern between experimental groups (Figure 4B), where ubiquitin expression was similar between young controls (CTRL < 6 M) and old LCPUFA-treated (LCPUFA 15 M) animals, but significantly different from old control animals (CTRL 15 M, *p* < 0.05). Noticeably, two-way ANOVA revealed strong age*diet interactions in the expression of 75 kDa η_p_^2^ = 0.182) and 150 kDa (η_p_^2^ = 0.166) as well as for the pooled variable (η_p_^2^ = 0.162) bands, which is in line with the effects of LCPUFA treatment described below. Finally, we observed that the LCPUFA/CTRL ratio for ubiquitin expression was notably higher in older animals than in young littermates, suggesting an age-dependent modulatory effect of LCPUFA (Figure 4C). Altogether, these results suggest an age- and diet-related modification of Nrf2 and Keap1 protein ubiquitination. Obviously, these results require further demonstration using more specific methods.

Current views suggest that under basal conditions the levels of Nrf2 protein are kept low by the E3 ubiquitin ligase Keap1, which ubiquitinates Nrf2 and targets it for degradation. Such constitutive degradation of Nrf2 allows for the basal expression of housekeeping target genes. Keap1 functions as a critical sensor of cellular stress. Its activity diminishes under conditions of oxidative stress or in the response to electrophilic xenobiotics. In these circumstances, Nrf2 can accumulate in the nucleus to activate the expression of inducible target genes. The high redox sensitivity of Keap1 is determined by the high number of cysteine residues (27 in the full-length human protein) distributed throughout the Keap1 sequence, which are susceptible to oxidation or to covalent modification by electrophiles [6,41]. Notably, cysteine residues behave differently depending on the context, giving rise to the concept of a “cysteine code”. According to this model, residues Cys273 and Cys288 may be essential for Keap1 to control Nrf2 under basal conditions, whereas other Cys residues are specifically required for stress conditions and for sensing specific toxicants [6,58]. However, the “cysteine code” related to aging has not been explored.

### 3.4. Effects of Aging and Dietary n-3 LCPUFA Supplementation on Hippocampal Markers of Lipoxidation

Electrophilic lipids and Nrf2 have traditionally been correlated. Therefore, we decided to explore the signature of lipid peroxidation in the hippocampus of mice fed on the n-3 PUFA diet. PUFAs are highly prone to peroxidation as a result of their highly unsaturated chemical structure, which proceeds through both enzymatic and non-enzymatic pathways. Non-enzymatic peroxidation of n-3 and n-6 PUFAs results in the formation of a number of bioactive intermediates, ultimately producing chemically stable and highly electrophilic aldehydes. Reactive aldehydes may interact with protein residues by nucleophilic attack forming Michael adducts.

MDA is one of the best studied α-, β-unsaturated aldehydes derived from n-3 and n-6 PUFA. HNE is another derivative formed from n-6 PUFA. These aldehydes have been described as potentially cytotoxic and mutagenic in many cellular systems [59,60,61]. However, the current view is that these electrophiles, in particular HNE, exhibit hormetic behavior in their biological effects. Thus, these molecules can act as signaling molecules at physiological levels, often inducing up-regulation of different enzymes responsible for ROS detoxification and cell survival. However, the electrophile adducts may induce cytotoxic responses at high levels by generating covalent modifications of macromolecular complexes [18,59,60,62,63].

**Figure 5:**
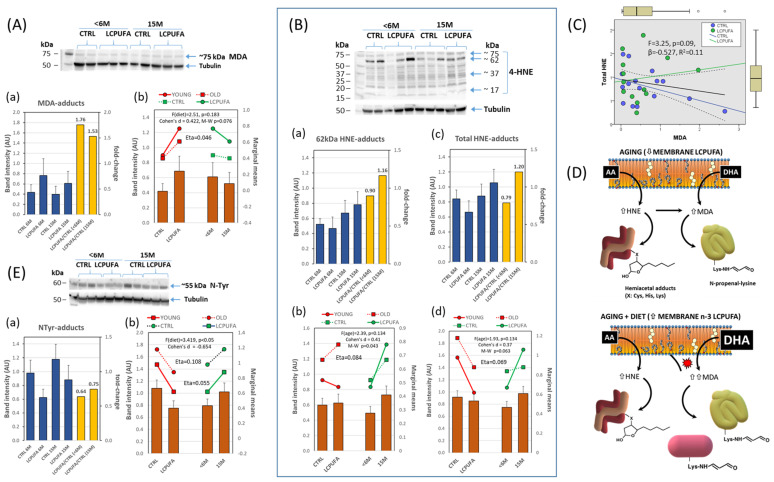
Formation of protein adducts in response to aging and dietary treatments. (**A**) Quantification of MDA adducts (**a**) in hippocampal extracts from the four groups (blue bars) along with the LCPUFA/CTRL ratios for different ages (yellow bars). Results from two-way ANOVA (**b**). A representative western blot for the different conditions is shown in the upper image. (**B**) Quantification of 62 kDa HNE (**a**) and total HNE (**c**) adducts in hippocampal extracts (blue bars) from the four experimental groups together with the LCPUFA/CTRL ratios for different ages (yellow bars). Results from two-way ANOVA (**b**,**d**). Representative western blot for the different groups is shown in the upper image. (**C**) Regression analyses for HNE and MDA adducts in the whole dataset and in pooled dietary groups. (**D**) Schematic representation of the formation of HNE and MDA adducts in aged hippocampus under normal and n-3 LCPUFA supplementation conditions. Aging itself is accompanied by membrane n-3 and n-6 LCPUFA, which is accompanied by increased lipoxidative compounds. DHA supplementation increases both membrane n-3 LCPUFA and the amount of n-3 LCPUFA-derived MDA, which is reflected in increased MDA adducts. (**E**) Quantification of N-Tyr adducts (**a**) in hippocampal extracts from the four groups (blue bars) along with the LCPUFA/CTRL ratios for different ages (yellow bars). Results from two-way ANOVA (**b**). A representative western blot for the different conditions is shown in the upper image. Number of samples per group: 7 (CTRL < 6 M), 8 (LCPUFA < 6 M), 7 (CTRL 15 M), 8 (LCPUFA 15 M).

We analyzed the presence of MDA adducts in the four experimental groups. The results shown in Figure 5A indicate no significant changes in the levels of MDA adducts between the four experimental groups. However, the LCPUFA/CTRL ratios for each age suggested an average 64.5% increase in MDA adducts in response to the diet (Figure 5(Aa)), which was similar between younger and older animals. The results from two-way ANOVA revealed an incipient effect of diet independent of age, which approached statistical significance (F = 2.51, *p* = 0.138; η_p_^2^ = 0.046). Then, we pooled together age groups in CTRL and LCPUFA subsets and performed non-parametric comparisons and the estimation of size effects. The Mann–Whitney test revealed a difference with *p* < 0.1 for the effect of diet, and a Cohen’s d value of +0.422 (Figure 5(Ab)), confirming a moderate positive effect of diet, rather than age, on the formation of MDA adducts.

Next, we determined HNE adducts in whole hippocampal extracts as a parameter of the lipoxidative damage generated by aging. The detailed analyses of membrane blots revealed several immunoreactive bands with molecular weights of 75 kDa, 62 kDa (canonical band), 37 kDa, and 17 kDa. The existence of such different bands is not unexpected given the unspecific HNE nucleophile attack reaction with protein amino acids. In fact, there are two principal ways of modifying amino acid side chains by 4-hydroxynonenal: via a Schiff’s base formation due to the reaction of the aldehydic group of HNE with an amino group of a protein (specifically Lys, His, Cys) and via a Michael addition of the HNE double bond to a protein side chain [61,64].

The analyses of HNE adducts were performed on the 62 kDa band and on the total HNE adducts. The results shown in Figure 5B indicate no significant differences between groups for any type of HNE adduct (Figure 5(Ba,c)). No effect was observed for the diet as analyzed with one-way ANOVA, although the age factor appeared to have moderated size effects (Figure 5(Bb,d)) with η_p_^2^ values of 0.084 and 0.069 in 62 kDa HNE and total HNE adducts. In fact, the LCPUFA/CTRL ratio increased by 0.26 and 0.41 fold in the 15 M group compared to the <6 M group, for 62 kDa HNE adducts and total HNE adducts, respectively (Figure 5(Ba,c)). Therefore, we pooled together diet groups with same age and analyzed group median differences and size effects. Hence, Kruskal–Wallis tests revealed a significant difference between the 15 M and <6 M groups in the 62 kDa band and also for the total HNE adduct, with Cohen’s d values of +0.41 and +0.37, respectively (Figure 5(Bd)), indicating an age-induced increase in HNE adducts without detectable interaction with the diet factor.

The apparent discrepancy in the results from the comparative study of MDA and HNE adducts might be explained by the fact that MDA adducts are formed from both n-3 and n-6 LCFUFA by-products. In contrast, HNE adducts derive from n-6 LCPUFA oxidation (especially from AA, the main n-6 fatty acid in nerve cells) [64,65]. Therefore, as only n-3 LCPUFA was supplied in the diet, we expected little or no effect from the diet on HNE adducts, but detectable changes in MDA adducts. Consequently, HNE adducts increased with aging independently of the diet.

Next, we performed regression analyses on the bivariate relationships between MDA and total HNE adducts (Figure 5C). The results using the whole dataset revealed a moderate positive correlation (*r* = 0.319, *p* < 0.1) with a regression coefficient (β) of 0.209 (F = 3.05, *p*= 0.09), indicating that MDA adducts increased in parallel with total HNE forms. However, this relationship drastically changed depending on the dietary condition, i.e., it vanished in LCPUFA groups (*r* = 0.005) but significantly increased to moderate-strong in CTRL groups with a correlation coefficient (*r* = 0.428, *p* < 0.05) and β values of 0.683 (F = 3.135, *p* < 0.1). Further, equivalent regression analyses for age groups indicated positive relationships for <6 M with significant slope (β = 1.078, F = 5.907, *p* = 0.03) and correlation coefficient (*r* = 0.559) compared to β = 0.184, F = 2.43 (*p* > 0.1) and r = 0.141 for the 15 M group.

Collectively, these results suggest that diet and age are influencing factors in setting the MDA/HNE adducts relationships. The aging parameter contributes mostly to MDA adducts formation in CTRL animals whereas DHA supplementation modulates n-3 LCPUFA oxidation, especially in older animals (depicted in Figure 5D). These findings may be explained by increased endogenous oxidative status in aged brains, as often reported in different cellular and rodent models as well as in human brains [3,66,67]. In agreement, in these same preparations, we have previously reported the age-dependent reduction of AA in hippocampal tissue [44]. Likewise, in *post-mortem* human frontal cortex and hippocampus, we have reported the reduction of LCPUFA in membrane rafts under non-pathological aging [68]. This is in consonance with increased HNE adducts in the same brain areas [69,70].

### 3.5. Effects of Aging and Dietary n-3 LCPUFA Supplementation on Hippocampal Markers of Nitrosylation

Following the effects of diet and age on lipoxidative damage, we next assessed the nitrosative status of hippocampal extracts by determining levels of NTyr protein adducts. Nitration of protein tyrosine residues is caused by the highly reactive peroxynitrite anion, (OONO^−^), which is formed by the reaction of superoxide anion (O_2_^−^) and nitric oxide (NO), in a rapid and stable process targeting surface residues, mainly tyrosine [71]. Although the nitrosative modification of proteins is linked to oxidative conditions through superoxide anion formation, it strongly dependent on NO generation [71].

The results in Figure 5(Ea) show significant differences between the CTRL and LCPUFA groups, with higher NTyr adduct levels found in CTRL groups at any age (*p* < 0.05, Cohen’s d = −0.65) and LCPUFA-to-CTRL ratios around 0.7. No significant effect was observed for the age factor on its own as analyzed by two-way ANOVA (Figure 5(Eb)). However, a degree of interaction between diet and age might underlie the slightly higher levels of NTyr adducts (and LCPUFA/CTRL ratio) in 15M animals compared to younger littermates (yellow bars in Figure 5(Ea)). Therefore, we concluded that dietary LCPUFA treatment promoted the control of RNS generation and nitrosative attack in hippocampal tissue in both young and older animals. Of note, the relationships between NTyr and MDA adducts were oppositely related between CTRL and LCPUFA groups (see Supplementary Figure S1), which suggests an LCPUFA-mediated regulation of NO generation, e.g., nitric oxide synthase, irrespective of oxidative stimulus.

The reduction of RNS in response to DHA treatments has been reported in different models of neurodegeneration and oxidative stress [3,72,73,74]. However, the present results provide the first direct demonstration of in vivo age-dependent LCPUFA modulation of protein nitration in hippocampal tissue in the absence of oxidative/nitrosative-induced insults, but in response to physiological age-associated changes [66,67,75].

### 3.6. Effects of Aging and Dietary n-3 LCPUFA Supplementation on Hippocampal Expression of HO-1 and GPx4

**Figure 6:**
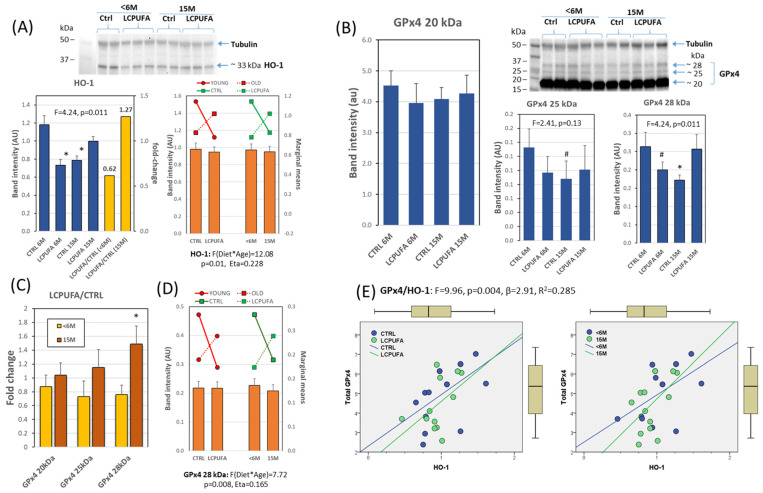
Target protein expression patterns in response to aging and dietary treatment. (**A**) Quantification of HO-1 in hippocampal extracts from the four groups (blue bars) along with the LCPUFA/CTRL ratios for different ages (yellow bars). Results from two-way ANOVA (right bar graph) for HO-1 showing the interaction between main factors. A representative western blot for the different conditions is shown in the upper image. * *p* < 0.05 compared to CTRL < 6 M. (**B**) Quantification of 20, 25, and 28 kDa GPx4 isoforms in hippocampal extracts from the four experimental group. #, * *p* < 0.1 and *p* < 0.05 compared to CTRL < 6 M. Representative western blot for the different groups is shown in the upper image. (**C**) LCPUFA-to-CTRL ratios for the three isoforms as a function of age. * *p* < 0.05 compared to <6 M. (**D**) Results from two-way ANOVA (right bar graph) for GPx4 28 kDa showing the interaction between main factors. (**E**) Regression analyses for total GPx4 and HO-1 in the whole dataset and in pooled dietary and age groups. Number of samples per group: 6 (CTRL < 6 M), 9 (LCPUFA < 6 M), 6 (CTRL 15 M), 9 (LCPUFA 15 M).

Finally, we determined the effects of Nrf2/Keap1 status on the expression patterns of two antioxidant target genes related to neuronal preservation in conditions of oxidative stress, i.e., inducible HO-1 and GPx4. The results summarized in Figure 6A reveal that HO-1 expression changes significantly between groups, with the lowest values found in LCPUFA < 6 M and CTRL 15 M groups, and the largest in CTRL < 6 M and LCPUFA 15 M. Noticeably, LCPUFA-to-CTRL ratios were much higher in the 15 M than in the <6 M groups indicating an age-dependent modulation of HO-1 expression by LCPUFA.

However, two-way ANOVA revealed no significant effects of the main factors, but a very strong interaction between age and diet in controlling HO-1 expression (Figure 6A, in the right panel). This complex response to dietary treatment as a function of age strongly suggests a neuroprotective role of LCPUFA against the increased oxidative conditions associated with aging [17,66,74,76]. In such a context, it is conceivable that dietary LCPUFA might promote the preservation of nerve cells in older individuals by limiting the effects of pro-oxidative redox status occurring during normal aging [3,66,77].

Furthermore, the reduced levels of HO-1 expression observed in young animals supplemented with LCPUFA were rather surprising. It is however known that HO-1 exhibits Janus-faced behavior and may lead to ‘core’ neuropathological features of degeneration (such as excessive deposition of non-transferrin bound iron, mitochondrial membrane damage, and macroautophagy) in nerve cells, especially in astrocytes [22,23,24]. The mechanism(s) for HO-1-induced neurotoxicity remain elusive, but it is accepted that they involve the accumulation of some by-products of heme catabolism, i.e., CO, ferrous iron and biliverdin/bilirubin [22,23].

In line with this, we may surmise that LCPUFA treatment activates a modulatory effect on HO-1 transcription in the hippocampus of young animals. In fact, besides Nrf2, the promotor region of the HO-1 gene contains binding sequences for different transcription factors such as NF-κB, hypoxia-inducible factor 1, and AP-1/2 [22], some of which might be regulated at some step in the transduction pathways activated by LCPUFA. This would prevent excessive HO-1 expression in animals where basal HO-1 is already elevated (Figure 6A), therefore hampering HO-1-derived by-products to initiate cytotoxic events. Additional experiments will be needed to confirm this hypothesis.

Next, we assessed the pattern of protein expression of phospholipid hydroperoxide GPx4. This phase II detoxifying enzyme is a selenoenzyme that plays a critical role in protecting nerve cell membranes under physiological, yet pro-oxidant, conditions of brain parenchyma [27,40,77]. It is unique among glutathione peroxidases in that it is capable of reducing complex phospholipid hydroperoxides in cell membranes, even without prior action of membrane phospholipase A2 [20,25].

The mammalian GPx4 family comprises three isoenzymes, which are found in the cytoplasm (c-GPx4), nucleus (n-GPx4), mitochondria (m-GPx4), and endoplasmic reticulum [20,26]. All these isoforms are very similar but differ in their N-terminal sequences. GPx4 isoenzymes derive from a single gene, *Gpx4*, by alternative splicing [25,26]. Interestingly, GPx4 expression and alternative splicing have been shown to be regulated by LCPUFA in hippocampal cells [36,40,78]. One main difference in transcriptional *Gpx4* regulation is that its three promotor regions lack canonical ARE sequences [25]. Therefore, its relationship with the Nrf2-Keap1 pathway has to be secondary to the activation of other transcriptional factors, such as AP1/2, SP1, or CREB [79], and indirectly linked to Nrf2 activation in response to lipid-derived aldehydes.

Here, we detected three GPx4 isoforms in western blots from hippocampal extracts migrating at 20 kDa, 25 kDa, and 28 kDa, likely corresponding to the cytosolic/mitochondrial and nuclear isoforms. It should be noted that the exact molecular weight of GPx4 isoforms may vary slightly between different studies due to technical/experimental conditions, and the post-translational modifications (such as ubiquitination, succination, phosphorylation, and glycosylation) [21].

In the present experimental paradigm, the 20 kDa isoform was predominant (average 91% of total GPx4) and displayed no expression changes in response to diet or aging (Figure 6B). Conversely, important changes could be detected for the higher molecular weight isoforms (25 kDa and 28 kDa), consisting of their expression reduction in aged control animals (Figure 6B) and augmented LCPUFA-to-CTRL ratios in older animals, particularly in the 28 kDa isoform (Figure 6C). This may be indicative of an age-dependent modulation of GPx4 expression by LCPUFA.

Two-way ANOVA revealed no significant effects of age and diet as the main factors but showed a very strong interaction between age and diet in controlling GPx4 expression (Figure 6D). This complex response to dietary treatment strongly suggests a compensatory role of LCPUFA in the decline of GPx4 28 kDa (and total GPx4) expression because of aging. The physiological significance of these findings is enormous because GPx4 is essential to rescue oxidized phospholipids in nerve cell membranes in situ, especially under conditions of mild to moderate oxidative environments [17,27]. Indeed, GPx4 is considered a neuroprotective enzyme against oxidative insults, and its depletion leads to cell death through ferroptosis [19,80].

Both the similar pattern of protein expression displayed by HO-1 and GPx4 isoforms across groups and the strong factor interaction between age and diet encouraged us to explore a potential relationship between the two protein variables. The results shown in Figure 6E demonstrate a positive linear association between HO-1 and total GPx4 in the whole dataset (*p* = 0.004). No apparent differences between CTRL and LCPUFA (left plot) or <6 M and 15 M (right plot) were observed in the regression analyses, as expected from the opposite factor interaction between main factors shown in Figure 6A,D. These results suggest that the increased expression of both enzymes might share similar regulatory mechanisms in response to aging and dietary interaction, at least in the hippocampal tissue.

### 3.7. Multivariate Associations between Nrf2, Keap1, HO-1, GPx4, and Lipoxidative/Nitrosative Variables in Response to Age–Diet Interactions

We finally used multivariate statistical approaches in order to assess the potential associations between Nrf2/Keap 1 complexes and the expression of HO-1 and GPx4 isoforms, in response to the different experimental conditions. First, we performed reliability analyses for internal consistencies of both the dataset and experimental groups. A Cochran’s alpha (Cα) value of 0.621 was obtained for the whole dataset (intraclass correlation F test = 2.823, *p* = 0.01). However, Cα changed significantly between groups (F = 120.78; *p* = 0.000). In fact, while CTRL < 6 M, LCPUFA < 6 M and LCPUFA 15 M groups exhibited high Cronbach’s alpha values (0.739, 0.671 and 0.668, respectively), the value observed for the CTRL 15 M group was substantially lower (−0.094) and not significant due to a negative average covariance among items, indicating a high intraclass variability, presumably related to different association mechanisms between Nrf2/Keap1/HO-1/GPx4 variables.

**Figure 7:**
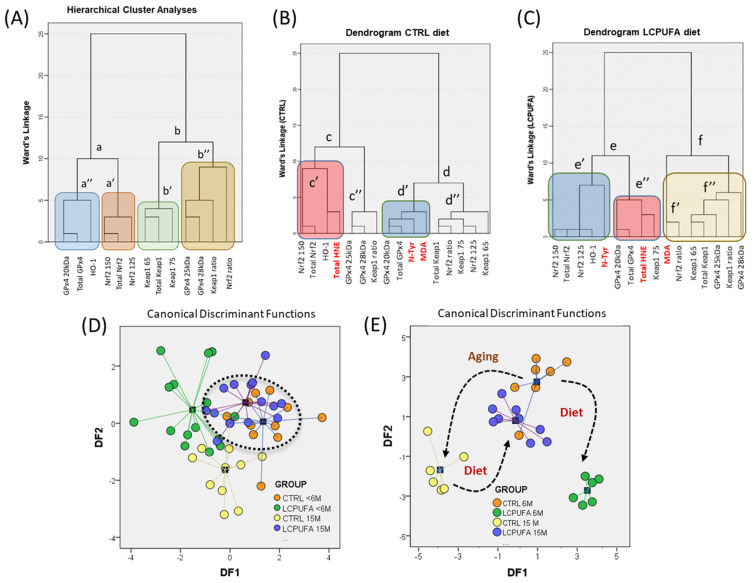
Multivariate analyses for the effects of age and diet factors on hippocampal Nrf2/Keap1 signaling. (**A**) Hierarchical cluster analyses (HCA) for Nrf2, Keap1, HO-1, and GPx4 variables in the whole dataset. Distances in the dendrograms were calculated using Ward’s linkage. Shadow rectangles highlight subclusters. (**B**,**C**) HCA for variables used in A plus incorporation of lipoxidative and nitrosative variables for CTRL (**B**) and LCPUFA (**C**) groups. Shadow rectangles highlight subclusters. Main clusters are indicated (a–f) along with their derived subclusters (a’, a”, b’, b”, c’, c”, d’, d”, e’, e”, f’, f”) (**D**) Linear discriminant function analyses (LDFA) for Nrf2, Keap1, HO-1, and GPx4 variables in the four nominal experimental groups. DF1 and DF2 explained 82.6% of the total variance. The dotted ellipse marks the highest proximity observed between the CTRL < 6 M and LCPUFA 15 M groups. (**E**) Incorporation of protein adducts to the analyses in D brought about a neat group discrimination. The reduced number of data points compared to the analyses in D results from the absolute requirement of LDFA to exclude cases containing missing values. Black arrows indicate transitional changes between groups as a consequence of the main factors.

Next, we performed hierarchical cluster analysis for variable linkage in the whole dataset (Figure 7A). The results revealed two clear proximity clusters (labelled **a** and **b**). In the first cluster, the expression of the most abundant GPx4 20kDa isoform and HO-1 were tightly associated in subcluster **a’**, in close proximity to the Nrf2 subcluster (**a”**). On the other hand, cluster **b** contained the higher molecular weight GPx4 isoforms (25 kDa and 28 kDa) and the Nrf2 and Keap1 ratios. Interestingly, Keap1 isoforms and total Keap1 associated as a separate subcluster (**b’**) within cluster **b**, as did Nrf2 in cluster **a**. This clustering pattern was essentially retained in individual groups, yet there were clear differences in the associations between members of the Nrf2/Keap1 protein set (Nrf2 150 kDa, Keap1 65 kDa and Keap1 75 kDa) and target antioxidant proteins (GPx4 28 kDa, GPx4 25 kDa and HO-1). Accordingly, it is assumable that besides changes in the expression of the transcription factor, aging modifies Nrf2/Keap1 signaling over regulated target genes, by virtue of a process that is modulated by dietary LCPUFA.

As it is expected that changes in Nrf2/Keap1 activity are mainly driven by signaling electrophiles, we next incorporated lipoxidative and nitrosative variables in the clustering analyses (Figure 7B). Under this paradigm, it is assumable that protein adducts are directly related to the generation/accumulation of corresponding reactive species (MDA, HNE, and peroxynitrite), which were analyzed in detail previously (Section 3.4 and Section 3.5). Accordingly, HCA were then performed on the CTRL and LCPUFA groups (Figure 7B). In the CTRL diet, we found two relevant associations, i.e., (1) a main linkage between HNE adducts and Nrf2 150/total Nrf2/HO-1 (subcluster **c’**) within cluster **c**, and (2) for GPx4 20 kDa/total GPx4/N-Tyr/MDA in subcluster **d’** of cluster **d**. On the other hand, in the LCPUFA diets, variable clustering changed so that the three electrophile-related adducts defined corresponding differentiated clusters. Thus, cluster **e** gathered together HO-1/Nrf2-x/N-Tyr in subcluster **e’**, and GPx4 20 kDa/total GPx4/HNE in subcluster **e”**. In the rightmost part of cluster **f**, MDA associated with LCPUFA-regulated GPx4 25 and 28 kDa isoforms, along with Keap1 variables.

These findings indicate that LCPUFAs (likely through MDA generation) regulate higher molecular weight GPX4 isoforms upon the modulation of the Keap1 isoform ratio in the Nrf2/Keap1 complex. It is also evident that NHE, which relates negatively to MDA (Figure 5), participates in Nrf2/Keap1-mediated HO-1 regulation (clusters **c’** and **e’**) and GPx4 20 kDa (clusters **d’** and **e”**), yet with different degrees of association between the CTRL and LCPUFA groups. Regarding N-Tyr adducts, no clear associations were detected for Nrf2 or Keap1, although an evident linkage was observed for GPx4r/total GPx4 in the CTRL and HO-1 in the LCPUFA groups. This agrees with the absence of significant bivariate relationships with aldehyde adducts (Section 3.5) and its low eigenvalues within canonical functions (see below).

The interpretation of these findings is that age-associated changes to LCPUFA-regulated HO-1 and GPx4 isoforms occur through the Nrf2 125 kDa/Keap1 75 kDa complex, yet Keap1 heterodimers cannot be disclosed, which indeed agrees with the observed Nrf2/Keap1 associations of Figure 3. The separate DLG and ETGE motifs in Nrf2 allow a single Nrf2 molecule to bind to the two Kelch domains present in the Keap1 dimer. These differences are thought to be important for fine-tuning regulation of the Nrf2-mediated stress response. A mechanism has been proposed in which the high-affinity ETGE motif acts as a hinge anchored to the first Kelch domain, whereas the weaker DLG motif is engaged as the latch to maintain minimal housekeeping expression levels [6].

Next, we performed LDFA. The results are summarized in Figure 7C. Three discriminant canonical functions were obtained, of which DF1 and DF2 displayed eigenvalues above 1, and explained 82.6% of total variance (50.2% and 32.4%, respectively). The analyses indicated high canonical correlations for DF1 (0.751) and DF2 (0.645). Variables with the largest absolute correlation between each variable and any DF were HO-1 > GPx4 28 kDa > Keap1 ratio > GPx4 25 kDa > Nrf2 150 > Keap75 for DF1 and Nrf2 ratio for DF2. Classification results indicated that 67.4% of original cases were correctly classified and 57.7% were cross-validated using the leave-one-out method [49]. Noticeably, the plotting of DFs showed a high degree of overlapping between the old LCPUFA and young CTRL groups (Figure 7C, dotted ellipse). However, the CTRL 15 M group was sufficiently differentiated as to appear in a separated cluster with the lowest DF2 scores, which is in agreement with the idea that aging brings about a decline in Nrf2/Keap1 signaling. This is very relevant since it may indicate that LCPUFA treatment rejuvenates the Nrf2/Keap1 complex profile found in old control animals (CTRL 15 M), to approach values found in young control animals (CTRL < 6 M), therefore partially restoring a young phenotype.

This latter hypothesis was further confirmed by the inclusion of protein adducts in the LDFA. The results in Figure 7D clearly show not only a lower dispersion in intra-group data, according to the proximity matrixes. They also reflect a neat segregation of experimental groups, especially those which appeared most distinct in the analyses summarized in 6C, i.e., CTRL 15M and LCPUFA < 6 M. However, young controls and old LCPUFA-treated remained closely grouped, reinforcing the restoration effect of LCPUFAs. The analyses indicated that the three DFs had eigenvalues above 1, high canonical correlations for DF1 (0.955), DF2 (0.913), and DF3 (0.738), and the first two DFs explaining 92.8% of the total variance. The inspection of protein adduct variables within the first two DFs indicated that N-Tyr was highly correlated to DF1 (along with HO-1 and Nrf2 125 kDa), while HNE and MDA adducts were highly determinant in defining DF2 (along with the Keap1 ratio: HO-1 > GPx4 28 kDa > Keap1 ratio > GPx4 25 kDa > Nrf2 150 > Keap75 for DF1 and Nrf2 ratio for DF2). Classification data indicated that 96.4% of original cases were correctly classified and 57.7% upon cross-validation.

**Figure 8:**
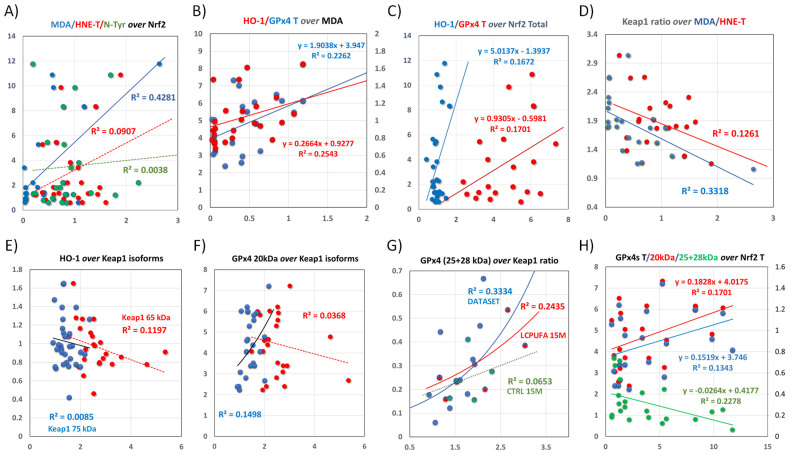
Bivariate relationships between main contributors to total variance from LDFA described in the previous multivariate analyses. (**A**) Regression lines for protein adducts and total Nrf2. Dotted lines indicate insignificant relationships (*p* > 0.1 in regression ANOVA). Only MDA adducts were significantly related to Nrf2. (**B**) Regression lines for target proteins HO-1 (red) and total GPx4 (blue), over MDA adducts. Regression equations are indicated for each subgroup. (**C**) Regression lines for target proteins HO-1 (blue) and total GPx4 (red) over total Nrf2. (**D**) Regression lines for Keap1 ratio and MDA (blue) or total HNE (red). (**E**,**F**) Regression analyses for the relationships between target proteins HO-1 (**E**) or HO-1 (**F**) and Keap1 isoforms. (**G**) Detailed regression analyses for GPx4 25 + 28 kDa and Keap1 ratio, in CTRL 15 M (green) and LCPUFA 15 M (red) groups, as well as for the whole dataset (blue). The exponential line in the whole dataset was chosen because it exhibited the highest correlation coefficient. (**H**) Regression analyses for the relationships between GPx4 proteins and total Nrf2. Regression equations are indicated for each GPx4 isoform. The effect size (R^2^) of regression outcomes is indicated in all plots.

We further explored the bivariate relationships of specific associations between variables in the sequence: reactive species ⇨ Nrf2/Keap1 structure ⇨ HO-1/GPx4 expression. Some of the results are shown in Figure 8 along with the magnitude of their effect sizes (R^2^). Following are the interpretations of these analyses. First, we detected a strong positive effect of MDA (but not total HNE or N-Tyr) on total Nrf2 (mainly through Nrf2 150 kDa) (Figure 8A). This finding was paralleled by a positive relationship between MDA and target genes HO-1 and total GPx4 (Figure 8B), and a moderate positive association between target proteins and total Nrf2 (Figure 8C). Therefore, it may be concluded that increased MDA (or some MDA-derived aldehyde such HHE) activates HO-1 and GPx4 expression through increased availability of Nrf2 150 kDa (recall that MDA levels increased in response to LCPUFA treatment, irrespective of animal age, as shown in Figure 5A).

In agreement with this notion, we have previously demonstrated in mouse hippocampal cells that DHA induces a time- and concentration-dependent generation of HHE, a reactive aldehyde specifically derived from DHA [36,78]. Paralleling this effect, DHA also stimulates the protein expression and enzyme activity of members of the glutathione and thioredoxin AOXs [36], most of which are encoded by genes containing canonical ARE sequences in their promotor regions [8,9,11].

Second, the Keap1 ratio (rather than individual isoforms or total Keap1) strongly associates with MDA and, to a lower extent, with total HNE adducts (Figure 8D), indicating that the amount of Keap1 65 kDa is reduced, while Keap1 75 kDa is increased in the formation of the Nrf2/Keap1 complex in response to aging and dietary treatment. The analyses of the dependence of HO-1 and GPx4 20 kDa expression on Keap1 isoforms indicate that the expression of HO-1 is negatively related to Keap1 65 kDa (Figure 8E), while that of GPx4 20 kDa is positively linked to Keap1 75 kDa (Figure 8F). These differences in the participation of Keap1 isoforms are likely responsible for the different clustering of GPx4 20 kDa (and total GPx4) and HO-1 shown in Figure 8B,C, despite the fact that HO-1 and total GPx4 are positively associated, independent of dietary treatment or age (Figure 6E).

Moreover, LCPUFA treatment increases the ratios of both Keap1 65 kDa and Keap1 75 kDa to Nrf2 150 kDa (Figure 3D) but reduces the same ratios in aged animals (Figure 3C). It may be concluded that the differences in the composition of Keap1/Nrf2 complexes underlie the correlated expression levels of both target proteins, especially in older animals. In addition, MDA adducts are increased in response to diet (Figure 5A) while for total HNE adducts, the LCPUFA-to-CTRL ratio was larger for LCPUFA-treated older animals (Figure 5C); suggesting that MDA mediates LCPUFA signals on the full-length Keap1 electrophile sensor [9,18,60], while HNE does so through the smaller Keap1 isoform.

Under these premises, the expression of the two target HO-1 and GPx4 proteins under unstimulated conditions may be promoted by MDA and HNE, in correlation with changes in the ratio of Keap1 isoforms in the complex with Nrf2 150 kDa.

Third, the paradoxical stimulation of higher molecular weight GPx4 isoforms by LCPUFAs observed in older animals as compared to young littermates (Figure 6B,C) is clearly linked to differential proportions between Keap1 isoforms. Indeed, the results in Figure 8G demonstrate the strong effect size for Keap1 75 kDa on the expression of the higher molecular weight GPx4 (25 + 28 kDa) isoforms (blue line). This finding was entirely attributed to LCPUFA induction (red line in Figure 8G) as it was neither observed in the CTRL diet (green dotted line) nor detected for the Keap1 65 kDa isoform.

Finally, the analyses of the dependence of GPx4 isoforms on the expression of Nrf2 indicate that the stimulation of higher molecular weight GPx4 isoforms was rather independent of the total Nrf2 amount (slope = 0.026). Opposite to this, GPx4 20 kDa and total GPx4 slopes were significantly positive (0.183 and 0.152, respectively). We concluded that the stimulatory effect of LCPUFAs of the higher molecular weight GPx4 proteins is triggered by the specific action of MDA on the fully functional Keap1 75 kDa, rather than by changes in the total amount of Keap1/Nrf2 complex.

In agreement, in previous studies on hippocampal HT22 cells, we found that DHA exposure induced the transcriptional activation of the *Gpx4* gene as well as its post-transcriptional splicing, leading to augmented c-*Gpx4* > m-*Gpx4* > n-*Gpx4*mRNAs. In the present context, this is likely to correspond to GPx4 20 kDa and higher molecular weight isoforms, respectively [36,40]. Interestingly, in the hippocampus of C57BL/6 mice exposed to low- and high-DHA diets, the transcriptional activation of *Gpx4* isoforms was highest for c-*Gpx4* on a low-DHA diet, a condition in which oxidative degradation of membrane DHA (and generation of reactive lipoxidative species) is favored [40,78].

**Figure 9:**
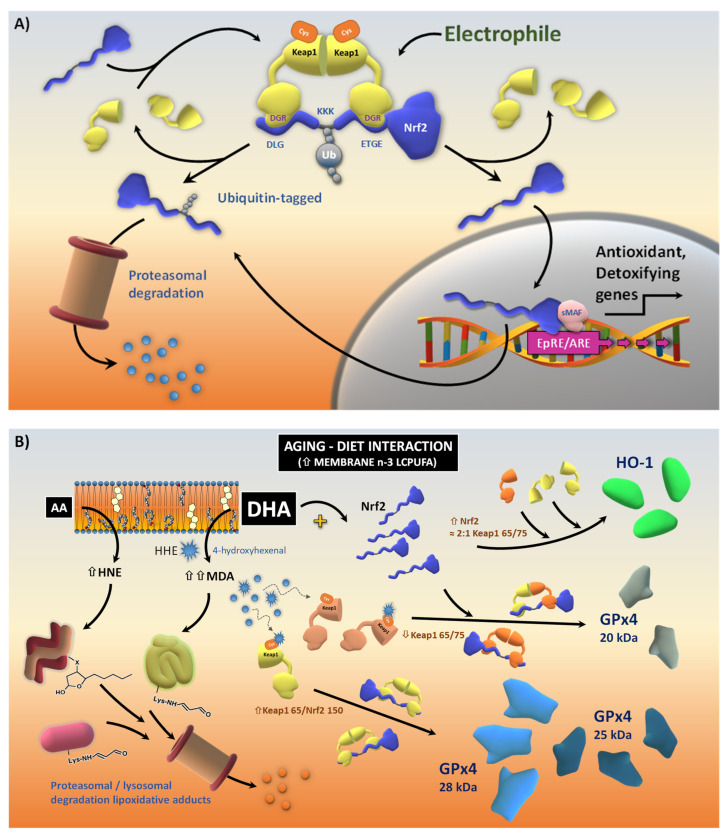
Models of Nrf2/Keap1 pathway regulation. (**A**) Canonical regulation. Under unstressed conditions, Nrf2 is polyubiquitinated through the Keap1/Cul3/Rbx1–E3 and degraded by the ubiquitin (Ub) proteasome pathway. The Keap1 dimer binds to Nrf2 molecules through the two-site DLGex and ETGE motifs flanking the K-rich ubiquitination motif. A small portion of Nrf2 accumulates in the nucleus and mediates the basal expression of ARE-dependent genes allowing basal maintenance of redox homeostasis under unstimulated conditions in response to basal generation of lipoxidative end-products (i.e., HNE, HNE, MDA) in nerve cells. Under oxidative stress conditions increased formation of electrophiles favor the reaction with cysteine codes in Keap1 to inhibit the E3 ligase activity of CUL3 and the polyubiquitination of Nrf2, resulting in the activation of Nrf2, its nuclear translocation, binding to sMAF, and the induction of ARE-dependent antioxidant/detoxifying genes. (**B**) The presence of higher membrane levels of DHA modifies the dynamics of Keap1/Nrf2/ARE pathway in two main directions, first by stimulating Nrf2 expression, and second by increasing intracellular electrophile messengers. Thus, in the pro-oxidant environment of hippocampal tissue, a significant fraction of DHA is non-enzymatically transformed to MDA (and likely HHE). These end-products react with different cysteine residues within the cysteine code and activate specific combinations of Keap1 isoforms, modifying the Keap1/Cul3/Rbx1–E3 ubiquitination activity on Nrf2. Unlike HO-1, whose stimulation by DHA is mainly related to the amount of Nrf2, the regulation of GPx4 isoforms is highly dependent on the cysteine code combination in Keap1 isoforms. Other transcriptional factors, such as PPAR-α/γ or NF-kB, might participate in the differential expression of GPx4 isoforms and other antioxidant/detoxifying genes lacking canonical ARE regions, but are stimulated in a Keap1/Nrf2-dependent way.

Finally, given the physiological relevance of dietary n-3 LCPUFA supplementation in aging, we have created a visual integration of the results from bivariate and multivariate analyses (Figure 9). In this sketch, hippocampal DHA triggers changes in the expression of Nrf2 subunits and in the proportions of Keap1 isoforms. Some of these effects are signaled through the generation of MDA (and likely HHE) which differentially activates Keap1 isoforms by binding to Cys residues at the Keap1 surface. A “cysteine code” might underlie the differential affinity of Keap1 isoforms for the reactive lipid aldehydes in older animals. Hence, the Keap1 dimer conformation would determine the degree of transcriptional activation of specific target genes.

Additional research aimed at testing the different steps and aspects in this alternative Keap1/Nrf2/ARE regulatory pathway is being carried out in our laboratories. It is also necessary to check for the presence and significance of this pathway in other models of aging, including the human hippocampus.

## 4. Conclusions

Overall, our results demonstrate the existence of modulatory mechanisms triggered by LCPUFAs on the cellular dynamics of the Keap1/Nrf2 signaling pathway, even in the absence of acute or persistent oxidative stress. Such mechanisms are subjected to physiological decline in response to normal aging but may be tuned to restore a more functional state, or “rejuvenated”, in response to dietary LCPUFA. At least in part, the effects of LCPUFAs are mediated by the generation of reactive lipid by-products, which may act as physiological intracellular messengers for oxidative response by adapting the composition of Keap1 dimers and downstream antioxidant gene transcription [59,62]. We believe that this research might have translational relevance as part of the nutraceutical strategies aimed to alleviate the cognitive decline associated with aging, or even the cognitive impairment related to some neurodegenerative pathologies.

## Figures and Tables

**Table 1:** Correlation matrixes for the Nrf2-Keap1 system. (**A**) Pearson’s and partial correlations for the whole dataset. (**B**) Pearson’s correlations as a function of diet (CONTROL vs LCPUFA). (**C**) Pearson’s correlations as a function of age (<6 M vs. 15 M).

**(A)**		**Partial Correlations (Control Variables AGE&DIET)**									
**Correlations** **DATASET**		**Keap1-65kDa**	**Keap1-75kDa**	**Keap1 65/75**	**Keap1 Total**	**Nrf2 150 kDa**	**Nrf2 125 kDa**	**Nrf2 125/150**	**Nrf2 Total**	**Keap1 65/** **Nrf2 150**	**Keap1 75/** **Nrf2 150**
**Pearson’s correlation**	**Keap1-65kDa**		0.335	**0.431**	**0.640**	**−0.440**	**−0.466**	0.280	**−0.484**	**0.498**	**0.440**
	**Keap1-75kDa**	**0.450**		**−0.512**	**0.615**	**0.443**	0.193	−0.267	**0.454**	−0.309	−0.261
	**Keap1 65kDa/75kDa**	**0.529**	−0.351		0.113	**−0.761**	**−0.525**	**0.473**	**−0.709**	**0.622**	**0.552**
	**Keap1 Total**	**0.774**	**0.634**	0.321		−0.060	−0.229	−0.007	−0.090	0.084	0.126
	**Nrf2 150 kDa**	**−0.440**	0.290	**−0.742**	−0.223		**0.639**	**−0.693**	**0.924**	**−0.726**	**−0.740**
	**Nrf2 125 kDa**	**−0.416**	0.070	**−0.484**	−0.275	**0.690**		−0.136	**0.668**	**−0.476**	**−0.468**
	**Nrf2 125/150**	0.294	−0.237	**0.426**	−0.024	**−0.552**	−0.074		**−0.616**	**0.809**	**0.839**
	**Nrf2 Total**	**−0.406**	**0.376**	**−0.682**	−0.151	**0.928**	**0.701**	**−0.495**		**−0.689**	**−0.695**
	**Keap1 65/Nrf2 150**	0.351	−0.261	**0.538**	0.070	**−0.610**	**−0.458**	**0.796**	**−0.608**		**0.971**
	**Keap1 75/Nrf2 150**	0.266	−0.245	**0.464**	0.057	**−0.613**	**−0.460**	**0.807**	**−0.604**	**0.980**	
**(B)**		**Diet: LCPUFA**									
**Correlations** **DIET**		**Keap1-65kDa**	**Keap1-75kDa**	**Keap1 65/75**	**Keap1 Total**	**Nrf2 150 kDa**	**Nrf2 125 kDa**	**Nrf2 125/150**	**Nrf2 Total**	**Keap1 65/** **Nrf2 150**	**Keap1 75/** **Nrf2 150**
**Diet: CONTROL**	**Keap1-65kDa**		0.415	**0.591**	**0.891**	**−0.578**	**−0.566**	**0.609**	**−0.546**	0.382	0.296
	**Keap1-75kDa**	**0.524**		−0.294	**0.685**	0.185	0.037	−0.253	0.268	−0.288	−0.280
	**Keap1 65kDa/75kDa**	0.402	−0.469		0.449	**−0.780**	**−0.538**	**0.603**	**−0.683**	**0.551**	**0.490**
	**Keap1 Total**	**0.656**	**0.593**	0.120		−0.394	−0.353	0.351	−0.325	0.168	0.125
	**Nrf2 150 kDa**	−0.145	**0.685**	**−0.727**	0.228		**0.728**	**−0.785**	**0.908**	**−0.699**	**−0.691**
	**Nrf2 125 kDa**	−0.167	0.160	−0.357	−0.093	0.390		−0.377	**0.748**	**−0.589**	**−0.574**
	**Nrf2 125/150**	−0.030	−0.244	0.216	−0.245	−0.429	0.331		**−0.703**	**0.882**	**0.914**
	**Nrf2 Total**	−0.156	**0.670**	**−0.732**	0.207	**0.995**	0.479	−0.373		**−0.676**	**−0.662**
	**Keap1 65/Nrf2 150**	0.277	−0.290	**0.623**	−0.074	**−0.680**	−0.383	**0.612**	**−0.689**		**0.985**
	**Keap1 75/Nrf2 150**	0.179	−0.240	**0.449**	−0.033	**−0.624**	−0.355	**0.655**	**−0.633**	**0.959**	
**(C)**		**Age: 15M**									
**Correlations** **AGE**		**Keap1-65kDa**	**Keap1-75kDa**	**Keap1 65/75**	**Keap1 Total**	**Nrf2 150 kDa**	**Nrf2 125 kDa**	**Nrf2 125/150**	**Nrf2 Total**	**Keap1 65/** **Nrf2 150**	**Keap1 75/** **Nrf2 150**
**Age: <6M**	**Keap1-65kDa**		**0.693**	**0.646**	**0.814**	**−0.513**	**−0.510**	0.044	**−0.520**	**0.613**	**0.527**
	**Keap1-75kDa**	0.162		−0.044	**0.697**	0.010	−0.057	−0.315	0.002	0.107	0.114
	**Keap1 65/75**	0.287	**−0.647**		**0.462**	**−0.680**	**−0.601**	**0.429**	**−0.680**	**0.717**	**0.596**
	**Keap1 Total**	**0.560**	**0.780**	−0.095		−0.358	**−0.462**	−0.254	−0.375	0.376	0.378
	**Nrf2 150 kDa**	**−0.503**	**0.448**	**−0.831**	−0.089		**0.874**	**−0.417**	**0.998**	**−0.701**	**−0.712**
	**Nrf2 125 kDa**	−0.361	0.280	**−0.555**	0.015	**0.748**		−0.090	**0.901**	**−0.605**	**−0.632**
	**Nrf2 125/150**	**0.580**	−0.210	0.467	0.198	**−0.607**	−0.088		−0.384	**0.754**	**0.720**
	**Nrf2 Total**	−0.310	**0.711**	**−0.767**	0.253	**0.894**	**0.448**	**−0.588**		**−0.699**	**−0.712**
	**Keap1 65/Nrf2 150**	0.343	**−0.448**	**0.595**	−0.118	**−0.617**	**−0.423**	**0.824**	**−0.593**		**0.971**
	**Keap1 75/Nrf2 150**	0.257	**−0.420**	**0.564**	−0.105	**−0.631**	**−0.424**	**0.856**	**−0.596**	**0.982**	

Number of cases in the analyses: 30 (Dataset), 13 (CTRL), 17 (LCPUFA), 15 (<6 M), 15 (15 M). Bold number indicate statistically significant differences (*p* < 0.05).

## Data Availability

The data that support the findings of this study are available on request from the corresponding author, M.D., and co-authors, C.V.B. and R.M.

## Supplementary Materials

The following supporting information can be downloaded at: https://www.mdpi.com/article/10.3390/antiox13020206/s1, Supplementary Table S1. Nutritive content of the standard and LCPUFA-enriched diets. Supplementary Figure S1. Correlation analyses for lipoxidative and nitrosative adducts. Upper matrix refers to Pearson’s and Partial correlations (controlling for age and diet) in the whole dataset. Middle and lower matrixes indicate Pearson’s correlation for main factors (age: middle matrix; diet: lower matrix).

## References

[1] Díaz M., Fabelo N., Casañas-Sánchez V., Marin R., Gómez T., Quinto-Alemany D., Pérez J.A. (2016). Hippocampal Lipid Homeostasis in APP/PS1 Mice Is Modulated by a Complex Interplay between Dietary DHA and Estrogens: Relevance for Alzheimer’s Disease. J. Alzheimers Dis..

[2] Rosenzweig E.S., Barnes C.A. (2003). Impact of Aging on Hippocampal Function: Plasticity, Network Dynamics, and Cognition. Prog. Neurobiol..

[3] Serini S., Calviello G. (2016). Reduction of Oxidative/Nitrosative Stress in Brain and Its Involvement in the Neuroprotective Effect of n-3 PUFA in Alzheimer’s Disease. Curr. Alzheimer Res..

[4] Yamamoto M., Kensler T.W., Motohashi H. (2018). The KEAP1-NRF2 System: A Thiol-Based Sensor-Effector Apparatus for Maintaining Redox Homeostasis. Physiol. Rev..

[5] Baird L., Yamamoto M. (2020). The Molecular Mechanisms Regulating the KEAP1-NRF2 Pathway. Mol. Cell. Biol..

[6] Canning P., Sorrell F.J., Bullock A.N. (2015). Structural Basis of Keap1 Interactions with Nrf2. Free Radic. Biol. Med..

[7] McMahon M., Itoh K., Yamamoto M., Hayes J.D. (2003). Keap1-Dependent Proteasomal Degradation of Transcription Factor Nrf2 Contributes to the Negative Regulation of Antioxidant Response Element-Driven Gene Expression. J. Biol. Chem..

[8] Niture S.K., Kaspar J.W., Shen J., Jaiswal A.K. (2010). Nrf2 Signaling and Cell Survival. Toxicol. Appl. Pharmacol..

[9] Ma Q., He X. (2012). Molecular Basis of Electrophilic and Oxidative Defense: Promises and Perils of Nrf2. Pharmacol. Rev..

[10] Hayes J.D., Dinkova-Kostova A.T. (2014). The Nrf2 Regulatory Network Provides an Interface between Redox and Intermediary Metabolism. Trends Biochem. Sci..

[11] Zhang H., Davies K.J.A., Forman H.J. (2015). Oxidative Stress Response and Nrf2 Signaling in Aging. Free Radic. Biol. Med..

[12] Levings D.C., Pathak S.S., Yang Y.-M., Slattery M. (2023). Limited Expression of Nrf2 in Neurons across the Central Nervous System. bioRxiv Prepr. Serv. Biol..

[13] Murphy T.H., Yu J., Ng R., Johnson D.A., Shen H., Honey C.R., Johnson J.A. (2001). Preferential Expression of Antioxidant Response Element Mediated Gene Expression in Astrocytes. J. Neurochem..

[14] Loboda A., Damulewicz M., Pyza E., Jozkowicz A., Dulak J. (2016). Role of Nrf2/HO-1 System in Development, Oxidative Stress Response and Diseases: An Evolutionarily Conserved Mechanism. Cell. Mol. Life Sci..

[15] Sykiotis G.P., Bohmann D. (2010). Stress-Activated Cap’n’collar Transcription Factors in Aging and Human Disease. Sci. Signal..

[16] Zhang D.D. (2006). Mechanistic Studies of the Nrf2-Keap1 Signaling Pathway. Drug Metab. Rev..

[17] Goldsteins G., Hakosalo V., Jaronen M., Keuters M.H., Lehtonen Š., Koistinaho J. (2022). CNS Redox Homeostasis and Dysfunction in Neurodegenerative Diseases. Antioxidants.

[18] Di Meo S., Reed T.T., Venditti P., Victor V.M. (2016). Role of ROS and RNS Sources in Physiological and Pathological Conditions. Oxid. Med. Cell. Longev..

[19] Cao J.Y., Dixon S.J. (2016). Mechanisms of Ferroptosis. Cell. Mol. Life Sci..

[20] Imai H., Nakagawa Y. (2003). Biological Significance of Phospholipid Hydroperoxide Glutathione Peroxidase (PHGPx, GPx4) in Mammalian Cells. Free Radic. Biol. Med..

[21] Cui C., Yang F., Li Q. (2022). Post-Translational Modification of GPX4 Is a Promising Target for Treating Ferroptosis-Related Diseases. Front. Mol. Biosci..

[22] Nitti M., Piras S., Brondolo L., Marinari U.M., Pronzato M.A., Furfaro A.L. (2018). Heme Oxygenase 1 in the Nervous System: Does It Favor Neuronal Cell Survival or Induce Neurodegeneration?. Int. J. Mol. Sci..

[23] Schipper H.M., Song W., Tavitian A., Cressatti M. (2019). The Sinister Face of Heme Oxygenase-1 in Brain Aging and Disease. Prog. Neurobiol..

[24] Waza A.A., Hamid Z., Ali S., Bhat S.A., Bhat M.A. (2018). A Review on Heme Oxygenase-1 Induction: Is It a Necessary Evil. Inflamm. Res..

[25] Ursini F., Bosello Travain V., Cozza G., Miotto G., Roveri A., Toppo S., Maiorino M. (2022). A White Paper on Phospholipid Hydroperoxide Glutathione Peroxidase (GPx4) Forty Years Later. Free Radic. Biol. Med..

[26] Savaskan N.E., Ufer C., Kühn H., Borchert A. (2007). Molecular Biology of Glutathione Peroxidase 4: From Genomic Structure to Developmental Expression and Neural Function. Biol. Chem..

[27] Savaskan N.E., Borchert A., Bräuer A.U., Kuhn H. (2007). Role for Glutathione Peroxidase-4 in Brain Development and Neuronal Apoptosis: Specific Induction of Enzyme Expression in Reactive Astrocytes Following Brain Injury. Free Radic. Biol. Med..

[28] Sambra V., Echeverria F., Valenzuela A., Chouinard-Watkins R., Valenzuela R. (2021). Docosahexaenoic and Arachidonic Acids as Neuroprotective Nutrients throughout the Life Cycle. Nutrients.

[29] Dyall S.C. (2015). Long-Chain Omega-3 Fatty Acids and the Brain: A Review of the Independent and Shared Effects of EPA, DPA and DHA. Front. Aging Neurosci..

[30] Bazinet R.P., Layé S. (2014). Polyunsaturated Fatty Acids and Their Metabolites in Brain Function and Disease. Nat. Rev. Neurosci..

[31] Marin R., Fabelo N., Fernández-Echevarría C., Canerina-Amaro A., Rodríguez-Barreto D., Quinto-Alemany D., Mesa-Herrera F., Díaz M. (2016). Lipid Raft Alterations in Aged-Associated Neuropathologies. Curr. Alzheimer Res..

[32] Karr J.E., Alexander J.E., Winningham R.G. (2011). Omega-3 Polyunsaturated Fatty Acids and Cognition throughout the Lifespan: A Review. Nutr. Neurosci..

[33] Yurko-Mauro K., Alexander D.D., Van Elswyk M.E. (2015). Docosahexaenoic Acid and Adult Memory: A Systematic Review and Meta-Analysis. PLoS ONE.

[34] Lee Y.Y., Galano J.M., Leung H.H., Balas L., Oger C., Durand T., Lee J.C.Y. (2020). Nonenzymatic Oxygenated Metabolite of Docosahexaenoic Acid, 4(RS)-4-F4t -Neuroprostane, Acts as a Bioactive Lipid Molecule in Neuronal Cells. FEBS Lett..

[35] Ishikado A., Nishio Y., Morino K., Ugi S., Kondo H., Makino T., Kashiwagi A., Maegawa H. (2010). Low Concentration of 4-Hydroxy Hexenal Increases Heme Oxygenase-1 Expression through Activation of Nrf2 and Antioxidative Activity in Vascular Endothelial Cells. Biochem. Biophys. Res. Commun..

[36] Casañas-Sánchez V., Pérez J.A., Fabelo N., Herrera-Herrera A.V., Fernández C., Marín R., González-Montelongo M.C., Díaz M. (2014). Addition of Docosahexaenoic Acid, but Not Arachidonic Acid, Activates Glutathione and Thioredoxin Antioxidant Systems in Murine Hippocampal HT22 Cells: Potential Implications in Neuroprotection. J. Neurochem..

[37] Borgonovi S.M., Iametti S., Di Nunzio M. (2023). Docosahexaenoic Acid as Master Regulator of Cellular Antioxidant Defenses: A Systematic Review. Antioxidants.

[38] Bang H.Y., Park S.A., Saeidi S., Na H.K., Surh Y.J. (2017). Docosahexaenoic Acid Induces Expression of Heme Oxygenase-1 and NAD(P)H:Quinone Oxidoreductase through Activation of Nrf2 in Human Mammary Epithelial Cells. Molecules.

[39] Zhang M., Wang S., Mao L., Leak R.K., Shi Y., Zhang W., Hu X., Sun B., Cao G., Gao Y. (2014). Omega-3 Fatty Acids Protect the Brain against Ischemic Injury by Activating Nrf2 and Upregulating Heme Oxygenase 1. J. Neurosci..

[40] Díaz M., Mesa-Herrera F., Marín R. (2021). DHA and Its Elaborated Modulation of Antioxidant Defenses of the Brain: Implications in Aging and AD Neurodegeneration. Antioxidants.

[41] Hur W., Gray N.S. (2011). Small Molecule Modulators of Antioxidant Response Pathway. Curr. Opin. Chem. Biol..

[42] Lee C. (2017). Collaborative Power of Nrf2 and PPAR γ Activators against Metabolic and Drug-Induced Oxidative Injury. Oxid. Med. Cell. Longev..

[43] Gendy A.M., El-Gazar A.A., Ragab G.M., Al-Mokaddem A.K., El-Haddad A.E., Selim H.M.R.M., Yousef E.M., Hamed N.O., Ibrahim S.S.A. (2022). Possible Implication of Nrf2, PPAR-γ and MAPKs Signaling in the Protective Role of Mangiferin against Renal Ischemia/Reperfusion in Rats. Pharmaceuticals.

[44] Taoro-González L., Pereda D., Valdés-Baizabal C., González-Gómez M., Pérez J.A., Mesa-Herrera F., Canerina-Amaro A., Pérez-González H., Rodríguez C., Díaz M. (2022). Effects of Dietary N-3 LCPUFA Supplementation on the Hippocampus of Aging Female Mice: Impact on Memory, Lipid Raft-Associated Glutamatergic Receptors and Neuroinflammation. Int. J. Mol. Sci..

[45] Cohen J. (1988). Statistical Power Analysis for the Behavioral Sciences.

[46] Sullivan G.M., Feinn R. (2012). Using Effect Size—Or Why the P Value Is Not Enough. J. Grad. Med. Educ..

[47] Lakens D. (2013). Calculating and Reporting Effect Sizes to Facilitate Cumulative Science: A Practical Primer for t-Tests and ANOVAs. Front. Psychol..

[48] Funder D.C., Ozer D.J. (2019). Evaluating Effect Size in Psychological Research: Sense and Nonsense. Adv. Methods Pract. Psychol. Sci..

[49] Huberty C.J. (1984). Issues in the Use and Interpretation of Discriminant Analysis. Psychol. Bull..

[50] Bryan H.K., Olayanju A., Goldring C.E., Park B.K. (2013). The Nrf2 Cell Defence Pathway: Keap1-Dependent and -Independent Mechanisms of Regulation. Biochem. Pharmacol..

[51] Ramani K., Tomasi M.L., Yang H., Ko K., Lu S.C. (2012). Mechanism and Significance of Changes in Glutamate-Cysteine Ligase Expression during Hepatic Fibrogenesis. J. Biol. Chem..

[52] Olejnik S., Algina J. (2003). Generalized Eta and Omega Squared Statistics: Measures of Effect Size for Some Common Research Designs. Psychol. Methods.

[53] Chen F., Xiao M., Feng J., Wufur R., Liu K., Hu S., Zhang Y. (2022). Different Inhibition of Nrf2 by Two Keap1 Isoforms α and β to Shape Malignant Behaviour of Human Hepatocellular Carcinoma. Int. J. Mol. Sci..

[54] Qiu L., Wang M., Zhu Y., Xiang Y., Zhang Y. (2018). A Naturally-Occurring Dominant-Negative Inhibitor of Keap1 Competitively against Its Negative Regulation of Nrf2. Int. J. Mol. Sci..

[55] Li L., Kobayashi M., Kaneko H., Nakajima-Takagi Y., Nakayama Y., Yamamoto M. (2008). Molecular Evolution of Keap1. Two Keap1 Molecules with Distinctive Intervening Region Structures Are Conserved among Fish. J. Biol. Chem..

[56] Kopacz A., Kloska D., Forman H.J., Jozkowicz A., Grochot-Przeczek A. (2020). Beyond Repression of Nrf2: An Update on Keap1. Free Radic. Biol. Med..

[57] Baird L., Llères D., Swift S., Dinkova-Kostova A.T. (2013). Regulatory Flexibility in the Nrf2-Mediated Stress Response Is Conferred by Conformational Cycling of the Keap1-Nrf2 Protein Complex. Proc. Natl. Acad. Sci. USA.

[58] Suzuki T., Muramatsu A., Saito R., Iso T., Shibata T., Kuwata K., Kawaguchi S.-i., Iwawaki T., Adachi S., Suda H. (2019). Molecular Mechanism of Cellular Oxidative Stress Sensing by Keap1. Cell Rep..

[59] Riahi Y., Cohen G., Shamni O., Sasson S. (2010). Signaling and Cytotoxic Functions of 4-Hydroxyalkenals. Am. J. Physiol. Endocrinol. Metab..

[60] Schopfer F.J., Cipollina C., Freeman B.A. (2011). Formation and Signaling Actions of Electrophilic Lipids. Chem. Rev..

[61] Castro J.P., Jung T., Grune T., Siems W. (2017). 4-Hydroxynonenal (HNE) Modified Proteins in Metabolic Diseases. Free Radic. Biol. Med..

[62] Finkel T. (2011). Signal Transduction by Reactive Oxygen Species. J. Cell Biol..

[63] Niki E. (2009). Lipid Peroxidation: Physiological Levels and Dual Biological Effects. Free Radic. Biol. Med..

[64] Sousa B.C., Pitt A.R., Spickett C.M. (2017). Chemistry and Analysis of HNE and Other Prominent Carbonyl-Containing Lipid Oxidation Compounds. Free Radic. Biol. Med..

[65] Esterbauer H., Schaur R.J., Zollner H. (1991). Chemistry and Biochemistry of 4-Hydroxynonenal, Malonaldehyde and Related Aldehydes. Free Radic. Biol. Med..

[66] Kregel K.C., Zhang H.J. (2007). An Integrated View of Oxidative Stress in Aging: Basic Mechanisms, Functional Effects, and Pathological Considerations. Am. J. Physiol. Regul. Integr. Comp. Physiol..

[67] Jomova K., Raptova R., Alomar S.Y., Alwasel S.H., Nepovimova E., Kuca K., Valko M. (2023). Reactive Oxygen Species, Toxicity, Oxidative Stress, and Antioxidants: Chronic Diseases and Aging. Arch. Toxicol..

[68] Díaz M., Fabelo N., Ferrer I., Marín R. (2018). “Lipid Raft Aging” in the Human Frontal Cortex during Nonpathological Aging: Gender Influences and Potential Implications in Alzheimer’s Disease. Neurobiol. Aging.

[69] Di Domenico F., Tramutola A., Butterfield D.A. (2017). Role of 4-Hydroxy-2-Nonenal (HNE) in the Pathogenesis of Alzheimer Disease and Other Selected Age-Related Neurodegenerative Disorders. Free Radic. Biol. Med..

[70] Butterfield D.A., Reed T., Perluigi M., De Marco C., Coccia R., Cini C., Sultana R. (2006). Elevated Protein-Bound Levels of the Lipid Peroxidation Product, 4-Hydroxy-2-Nonenal, in Brain from Persons with Mild Cognitive Impairment. Neurosci. Lett..

[71] Bandookwala M., Sengupta P. (2020). 3-Nitrotyrosine: A Versatile Oxidative Stress Biomarker for Major Neurodegenerative Diseases. Int. J. Neurosci..

[72] Clementi M.E., Lazzarino G., Sampaolese B., Brancato A., Tringali G. (2019). DHA Protects PC12 Cells against Oxidative Stress and Apoptotic Signals through the Activation of the NFE2L2/HO-1 Axis. Int. J. Mol. Med..

[73] Wang X., Zhao X., Mao Z.Y., Wang X.M., Liu Z.L. (2003). Neuroprotective Effect of Docosahexaenoic Acid on Glutamate-Induced Cytotoxicity in Rat Hippocampal Cultures. Neuroreport.

[74] Olufunmilayo E.O., Gerke-Duncan M.B., Holsinger R.M.D. (2023). Oxidative Stress and Antioxidants in Neurodegenerative Disorders. Antioxidants.

[75] Kelly L., Grehan B., Della Chiesa A., O’Mara S.M., Downer E., Sahyoun G., Massey K.A., Nicolaou A., Lynch M.A. (2011). The Polyunsaturated Fatty Acids, EPA and DPA Exert a Protective Effect in the Hippocampus of the Aged Rat. Neurobiol. Aging.

[76] Lee J., Kim H.J. (2022). Normal Aging Induces Changes in the Brain and Neurodegeneration Progress: Review of the Structural, Biochemical, Metabolic, Cellular, and Molecular Changes. Front. Aging Neurosci..

[77] Díaz M., Casañas-Sánchez V., Marín R., Pérez J.A., Díaz M., Casañas-Sánchez V., Marín R., Pérez J.A., Catalá A. (2017). Fighting against Lipid Peroxidation in the Brain: The Unique Story of Docosahexaenoic Acid. Lipid Peroxidation: Inhibition, Effects and Mechanisms.

[78] Casañas-Sánchez V., Pérez J.A., Fabelo N., Quinto-Alemany D., Díaz M.L. (2015). Docosahexaenoic (DHA) Modulates Phospholipid-Hydroperoxide Glutathione Peroxidase (Gpx4) Gene Expression to Ensure Self-Protection from Oxidative Damage in Hippocampal Cells. Front. Physiol..

[79] Imai H., Saito M., Kirai N., Hasegawa J., Konishi K., Hattori H., Nishimura M., Naito S., Nakagawa Y. (2006). Identification of the Positive Regulatory and Distinct Core Regions of Promoters, and Transcriptional Regulation in Three Types of Mouse Phospholipid Hydroperoxide Glutathione Peroxidase. J. Biochem..

[80] Xie Y., Hou W., Song X., Yu Y., Huang J., Sun X., Kang R., Tang D. (2016). Ferroptosis: Process and Function. Cell Death Differ..

