# Supplementary material for: Age-Dependent Changes in Nrf2/Keap1 and Target Antioxidant Protein Expression Correlate to Lipoxidative Adducts, and Are Modulated by Dietary N-3 LCPUFA in the Hippocampus of Mice
Source: Antioxidants (Basel). 2024 Feb 6;13(2):206. doi: 10.3390/antiox13020206 (PMC10886099; doi:10.3390/antiox13020206)

Supplementary Material

Supplementary Table S1. Nutritive content of the standard and LCPUFA-enriched diets.

Additives	Standard Diet (per kg)	LCPUFA-Enriched Diet (per kg)
**Vitamin A**	21000 (UI)	21000 (UI)
**Vitamin D_3**	1100 (UI)	1100 (UI)
**Iron**	50 mg	50 mg
**Magnesium**	40 mg	40 mg
**Zinc**	31 mg	31 mg
**Copper**	7 mg	7 mg
**Iodine**	6.2 mg	6.2 mg
**Technicall additives**	**Standard diet (per kg)**	**LCPUFA-enriched diet (per kg)**
**Sepiolite**	760 mg	760 mg
**Analytical constituents**	**Standard diet (%)**	**LCPUFA-enriched diet (per kg)**
**Moisture**	12.00	12.00
**Crude protein**	14.50	14.50
**Crude fibers**	4.50	4.50
**Crude ash**	4.70	4.70
**Crude oil and fats**	4.00	4.82
**Of which fatty acids**	**Standard diet (%)**	**LCPUFA-enriched diet (%)**
**C16:0 (Palmitic acid)**	0.5	0.5
**C18:0 (Stearic acid)**	0.1	0.1
**C18:1n9 (Oleic acid)**	0.7	0.7
**C18:2n6 (Linoleic acid)**	2.0	2.0
**C18:3n3 (Linolenic acid)**	0.1	0.1
**C20:5n3 (EPA)**	—	0.56
**C22:6n3 (DHA)**	—	0.26
**Total fatty acids classes**	**Standard diet (%)**	**LCPUFA-enriched diet (%)**
**Saturated (total)**	0.6	0.6
**Monounsaturated (total)**	0.7	0.7
**Polyunsaturated (total)**	2.1	2.92

Ingredients: Wheat middlings, ground wheat, ground corn, corn gluten meal, calcium carbonate, soybean oil, dicalcium phosphate, iodized salt, magnesium oxide

Supplementary Figure S1. Correlation analyses for lipoxidative and nitrosative adducts. Upper matrix refers to Pearson´s and Partial correlations (controlling for age and diet) in the whole dataset. Middle and lower matrixes indicate Pearson´s correlation for main factors (age: middle matrix; diet: lower matrix).

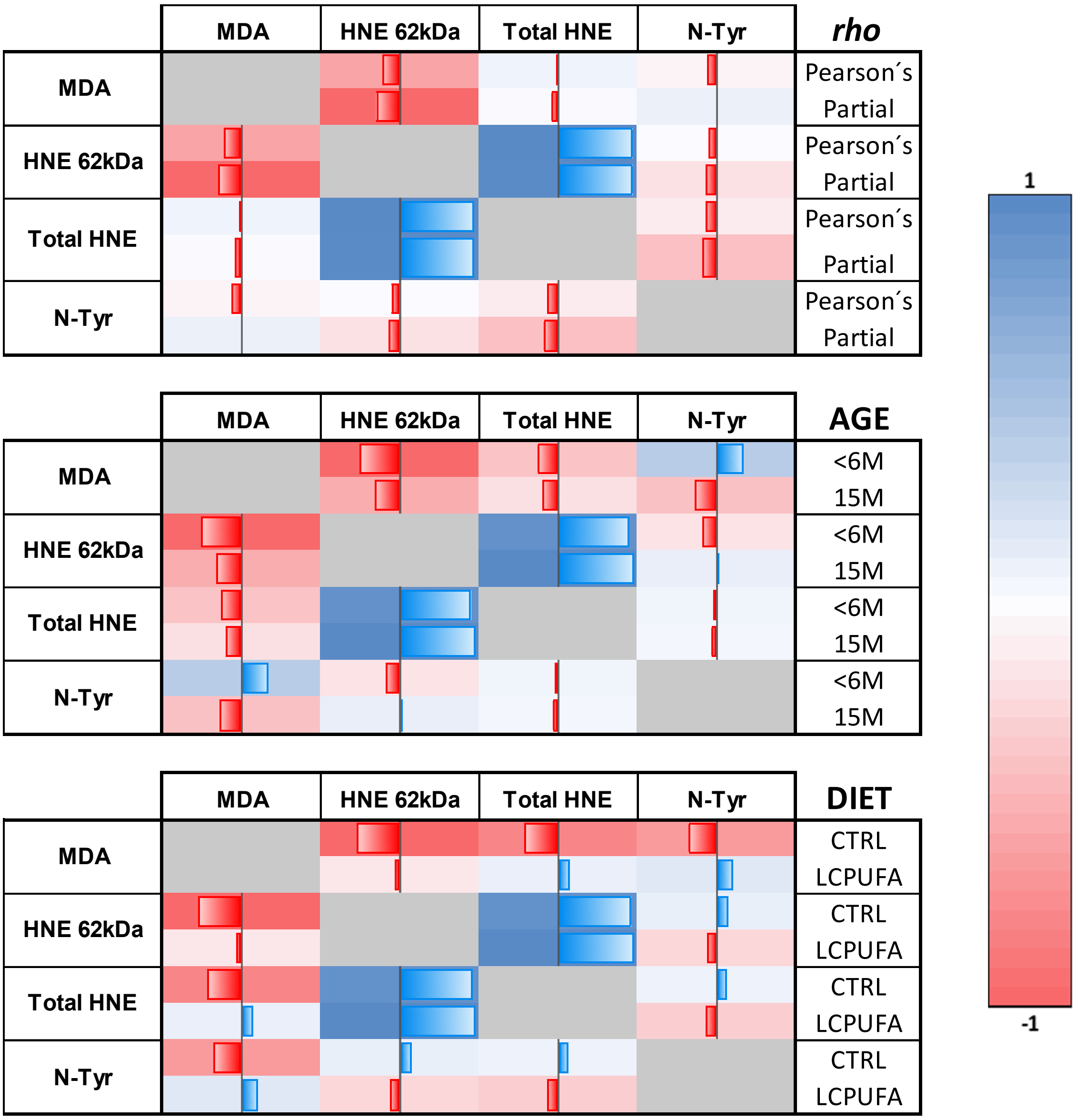

Supplement: Supplementary file 1 [file antioxidants-13-00206-s001.zip › antioxidants-2703919-supplementary.pdf]
